# Single-Molecule Electron Transport in Peptoids

**DOI:** 10.1021/acs.jpcb.5c07788

**Published:** 2026-03-04

**Authors:** Brittany Prempin, Rajarshi Samajdar, Hemani Chhabra, Moeen Meigooni, Aleksei Aksimentiev, Emad Tajkhorshid, Jeffrey S. Moore, Charles M. Schroeder

**Affiliations:** † Department of Chemistry, 14589 University of Illinois Urbana—Champaign, Urbana, Illinois 61801, United States; ‡ Beckman Institute for Advanced Science and Technology, 14589 University of Illinois Urbana—Champaign, Urbana, Illinois 61801, United States; § Department of Chemical and Biomolecular Engineering, 14589 University of Illinois Urbana—Champaign, Urbana, Illinois 61801, United States; ∥ Center for Biophysics and Quantitative Biology, 14589 University of Illinois Urbana—Champaign, Urbana, Illinois 61801, United States; ⊥ Department of Physics, 14589 University of Illinois Urbana—Champaign, Urbana, Illinois 61801, United States; # Department of Biochemistry, 14589 University of Illinois Urbana—Champaign, Urbana, Illinois 61801, United States; ∇ Department of Bioengineering, 14589 University of Illinois Urbana—Champaign, Urbana, Illinois 61801, United States; ○ Department of Materials Science and Engineering, 14589 University of Illinois Urbana—Champaign, Urbana, Illinois 61801, United States

## Abstract

Peptoids are structural analogs of peptides in which side chains are appended to the backbone nitrogen rather than the α-carbon. The sequence-defined modularity of peptoids enables precise control over structure–function relationships, enabling applications in energy storage and biomedical materials. Despite recent progress, the role of sequence and conformation on electron transport in peptoid molecules is not fully understood. Here, we synthesize a library of peptoid oligomers and characterize their molecular electronic properties using the scanning tunneling microscope-break junction (STM-BJ) technique. Our results show well-defined electron transport behavior for peptoid sequences containing aromatic side groups lacking hydrogen bonds (H-bonds) and without chemical substitutions at the N–C_α_ position. This behavior fundamentally differs from electron transport in peptides, where H-bond interactions give rise to higher conductance states. All-atom molecular dynamics (MD) simulations are used to understand the conformational heterogeneity of peptoids, and molecular conformations obtained from MD simulations are used in quantum mechanical calculations based on the nonequilibrium Green’s function–density functional theory (NEGF-DFT) formalism. In all cases, computational results are in reasonable qualitative agreement with experiments. Our work demonstrates that the conductance behavior of peptoids depends on monomer identity, including side-chain aromaticity and substitution at the N–C_α_ position. Overall, this work provides new insights into the structure–function relationships governing electron transport in peptoid-based materials and establishes design rules for peptoid-based molecular junctions.

## Graphical abstract

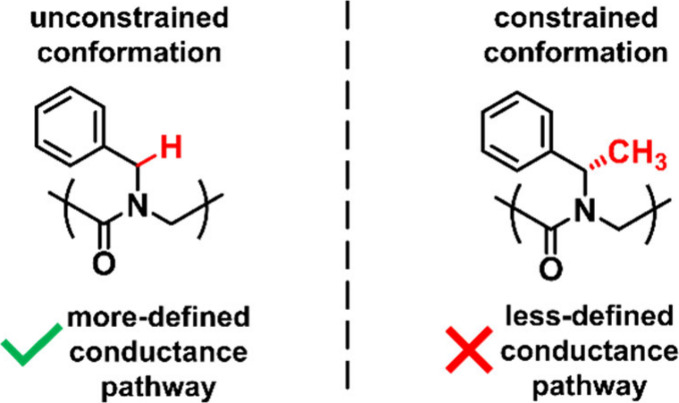

## Introduction

Electron transport in biomolecules is essential for a wide range of technological applications including molecular sensors, biomedical devices, and energy storage.^1–3^ In nature, electron transport is critical for maintaining fundamental life processes such as respiration and photosynthesis.^4^ In recent years, a combination of experiments and theoretical studies have focused on understanding the electronic properties of complex biological systems.^5–10^ Understanding electron transport at the molecular scale provides a useful framework for elucidating the sequence–structure–function relationships that govern chemically complex biomolecules.^11–15^ Recent work has shown that the electronic properties of short peptide oligomers critically depend on the conformational flexibility of the peptide backbone, with two distinct conductance states arising from either hydrogen bond (H-bond) mediated secondary structure or extended peptide conformations.^16^ Moreover, the molecular conductance behavior of peptides is not significantly affected by variation in sequence.^16^ Extending beyond natural biomolecules such as peptides, additional investigations are needed to understand the electron transport properties of bioinspired molecules such as peptoids.

Peptoids, or N-substituted polyglycine oligomers, are a class of bioinspired sequence-defined molecules that allow for precise control over structure–function relationships.^17,18^ Peptoids are promising materials for molecular electronic applications due to their diverse chemical space and modular design properties. Peptoids are structural analogs of peptides wherein the peptoid side chains are attached to the nitrogen atom of the backbone rather than the α-carbon.^19^ This key difference in chemical structure precludes backbone H-bonding interactions and enhances backbone flexibility compared to peptides.^20,21^ Prior work has shown that peptoids helical structures can have effective intramolecular energy transfer between side groups that are cofacially aligned.^22^ From this perspective, variations in side chains and substitutions at the N–C_α_ position are known to influence the structural and electronic properties of peptoids. Despite recent advances, we lack a complete understanding of electron transport in peptoids, particularly how local conformational properties and side-chain interactions influence transport.

In this work, we designed, synthesized, and characterized the electronic properties of a library of sequence-defined peptoid oligomers. Peptoid oligomers were synthesized using a two-step submonomer synthesis method that iteratively couples haloacetic acids to primary amines, enabling facile incorporation of non-natural side groups into peptoid backbones.^23^ Following synthesis and chemical characterization, the structural and electronic properties of peptoid oligomers were studied using a combination of molecular electronic techniques, molecular dynamics (MD) simulations, and quantum mechanical calculations. A scanning tunneling microscope break junction (STM-BJ) technique was used to experimentally characterize the molecular charge transport properties of sequence-defined peptoid oligomers. Our results show that peptoid sequences with aromatic side groups and without substitutions at the N–C_α_ position led to well-defined molecular conductance features. MD simulations are used to understand the conformational heterogeneity of peptoid backbones. Conformers generated from MD simulations are used in quantum mechanical calculations, and the results are in reasonable agreement with the experiments. Our results show well-defined electron transport pathways for peptoids with aromatic side groups lacking hydrogen bonds (H-bonds) and without chemical substitutions at the N–C_α_ position. This behavior contrasts with peptide oligomers, which display two-state conductance behavior characterized by a high-conductance feature associated with a folded conformation (stabilized by secondary structure from H-bonding interactions) and a low conductance state corresponding to an extended conformation. Overall, these results reveal new insights into the structure–function relationships of electron transport in peptoid oligomers.

## Methods

### Chemical Synthesis and Chemical Characterization

Peptoid oligomers were synthesized using a two-step submonomer synthesis method where the iterative coupling of haloacetic acids to primary amines is used to sequentially grow the chain.^23^ Our method deviates from the traditional use of bromoacetic acid in favor of chloroacetic acid to compatibilize the coupling conditions with the thio-methyl–containing submonomer amine (2-(methylthio)­ethylamine) (Scheme S1). Peptoid oligomers were synthesized manually using a Rink Amide resin (100–200 mesh, 0.78 mmol/g, Novabiochem) and commercially available submonomers based on previously reported procedures.^23^ The acetylation step used chloroacetic acid instead of bromoacetic acid because chloroacetic acid was found to be more compatible with the thio-methyl containing amine submonomer, 2-(methylthio)­ethylamine. All other conditions were used based on literature standards. Rink Amide resin (100 mg) was swelled in *N,N*-dimethylformamide (DMF) for 30 min and deprotected with piperidine (1 mL, 20% v/v in DMF). An acetylation reaction was performed by adding chloroacetic acid (1 mL, 0.4 M in DMF) and *N,N*-diisopropylcarbodiimide (DIC) (0.2 mL, 2 M in DMF). The mixture was agitated for 5 min, drained, and washed with DMF. Nucleophilic displacement was performed by adding the appropriate submonomer amine (1 mL, 2 M in *N*-methylpyrrolidone (NMP)) and mixing for 1 h (3 h for 2-(methylthio)­ethylamine). The acetylation and displacement steps were repeated until the peptoid of desired sequence is achieved. At the end of the synthesis, the resin was washed and dried with dichloromethane (DCM). Peptoids were cleaved from the resin using a trifluoroacetic acid (TFA) cleavage cocktail (95% TFA, 2.5% triisopropylsilane, 2.5% water) for 20 min. Three mL of cleavage cocktail was used for each 100 mg of resin. The crude solution was filtered out of the resin and the resin was rinsed with an additional 2 mL of cleavage cocktail and 5 mL of DCM. The crude mixture was dried using nitrogen flow in a Torviq Solvent Evaporator. The crude material was purified using a preparative Waters high pressure liquid chromatography (HPLC) system on a reverse-phase C18 column. The fractions were collected and lyophilized to yield the final product. The final products were analyzed using HR-ESI MS and analytical HPLC (Figures S1–S14). Following synthesis, high-resolution electrospray ionization mass spectrometry (HR-ESI MS) and analytical high-performance liquid chromatography (HPLC) were used to characterize the peptoid oligomers (Figures S1–S14). HR-ESI spectra were collected on a Waters Q-TOF Ultima ESI spectrometer. Analytical HPLC spectra were collected on a Shimadzu Prominence HPLC system with a reverse-phase C18 column. Circular dichroism (CD) spectroscopy measurements were collected on a Jasco J-1500 spectrophotometer at 298 K (Figure S15).

### Single-Molecule Conductance Measurements

Single-molecule conductance measurements were performed using a custom-built STM-BJ instrument, as previously reported.^16,24–28^ The STM-BJ setup consists of a gold tip electrode that is repeatedly moved into and out of contact with a gold substrate electrode in an aqueous solution containing a small concentration of the peptoids (peptoid concentration <1 mM), resulting in the continual formation and breakage of single-molecule junctions. Peptoid concentrations (<1 mM) were selected to yield Poisson statistics in molecular conductance traces. Gold STM tips were prepared using 0.25 mm Au wire (99.998%, Alfa Aesar). STM-BJ experiments were carried out in Corning cell culture grade water (product number 255–055-CV). Due to the polarity of the solvent, STM tips were coated with an Apiezon wax to prevent Faradaic currents from masking characteristic molecular features.^29^ Gold substrates for the measurements were prepared by evaporating 120 nm of gold on polished AFM metal discs (Ted Pella). Single-molecule conductance data were analyzed using one- and two-dimensional (1D and 2D) conductance histograms without data selection. In 1D conductance histograms, all recorded conductance values over the course of the measurement were compiled. The peak of the 1D conductance histogram corresponds to the most probable conductance value for a given molecule. 2D molecular conductance histograms show the distribution of conductance values together with junction separation distances, providing insights into the evolution of conductance as the junction is extended. All STM-BJ measurements on peptoids were performed in water at room temperature (298 K) at an applied bias of 250 mV with ensemble sizes of >5000 single molecules.

### Cluster Analysis and Gaussian Mixture Modeling

Unsupervised machine learning algorithms (such as k-means + +, spectral clustering, and Gaussian mixture modeling) have been previously used to analyze single-molecule charge transport data.^30^ Here, we employ silhouette score clustering in combination with Gaussian mixture modeling (GMM). GMM offers advantages over alternative methods such as k-means due to its ability to identify subpopulations with unequal covariance. This combined approach is commonly used to study bimodal conductance distributions or to identify the presence of multiple anchoring groups that give rise to distinct transport pathways in molecular-scale break junction experiments.^12,16,28,31^ Silhouette scores are calculated for obtaining the number of clusters across an ensemble of ∼ 5000 single molecule pulling traces.^32^ Silhouette scores indicate how similar a feature is to its own cluster as compared to another cluster. From each individual trace, a 30-by-30 two-dimensional histogram and a 100-bin one-dimensional histogram are determined which are then combined to form a 1000-dimensional feature space (30 × 30 + 100) over which GMM operates. The classification operates on the conductance range of 0 to −5.5 log­(*G/G*_*o*_), and displacement range of −0.1 to 1 nm (0 nm corresponds to the point where the junction is broken, the −0.1 to 0 nm regime indicates metal–metal contact. All traces are aligned at 0.5 *G*_*o*_ as the starting point of displacement).

### Molecular Dynamics Simulations

Molecular dynamics (MD) simulations were performed to generate conformational ensembles for the peptoid molecular junctions at three anchor displacements (referred to as stages 6 Å, 9 Å, and 12 Å). For each peptoid, 16 initial structures were prepared using the STEPs peptoid force field.^33^ Terminal caps were added, using NH for the N-terminus and NH_2_ for the C-terminus. The backbone dihedrals of each of the structures were randomized using tleap module in AMBER.^34^ Phi and psi backbone dihedral angles were randomized between −180 and 180 deg, whereas omega backbone dihedral angles were randomly set to either 0 or 180 deg. After backbone dihedral randomization, ring piercings were identified and mitigated using a novel approach involving removing nonbonded interactions between the atoms of the piercing bond and three atoms from the pierced ring (Figure S21). Peptoid structures were then solvated in a cubic box of TIP3P^35^ water of side length 40 Å using the tleap.^34^ The solvated systems were simulated using OpenMM 7.7.0.^36^ Dynamics were integrated using the LangevinMiddleIntegrator^37^ with friction coefficient of 1 ps^–1^, temperature of 300 K, and a time step of 2 fs. Bonds involving hydrogen atoms, and all bonds and angles involving water were constrained. Nonbonded interactions were computed with a cutoff of 12 Å with smooth switching starting at 10 Å. Electrostatic interactions were evaluated using particle mesh Ewald (PME) summation^38^ with error tolerance of 0.0005. Each replicate was simulated for 100 ns for each of three holding stages, for a total aggregate simulation time of 19.2 μs (4 peptoids × 3 stages × 16 replicates × 100 ns). Holding stages were enforced using a series of custom external potentials, applied using OpenMM’s custom potentials, described below. The last 90 ns of each simulation was used for subsequent analysis. Analysis and visualization were performed using MDAnalysis^39^ and VMD,^40^ respectively.

A series of custom potentials was implemented to implicitly represent interactions between the peptoid and gold particles as described in our prior work.^16^ Three potentials were defined: (1) a potential to restrain the distance between the anchors of the molecular junction along the pulling axis to 6 Å, 9 Å, or 12 Å (representing the restraints imposed by connections to the gold electrodes); (2) a per-atom charge-dependent potential along the pulling axis accounting for electric field forces arising from a voltage-biased junction; and, (3) a potential that orients N-methionine’s thioether moiety such that the average position of each anchoring sulfur’s lone pairs are oriented toward the (implicitly represented) gold electrodes along the pulling axis. For each peptoid, the conformation was selected from their 6 Å holding-stage simulations from the central distribution of S–S xy versus S–S z distance plots for electron transport calculations. All free energy plots (Supplementary Figures S22–23) were prepared using PyEMMA 2.5.11.^41^

### Principal Component Analysis (PCA) of MD Trajectories

The resulting MD trajectory data were subjected to dimensionality reduction by means of principal components analysis (PCA) (Figure S32, S33). For Nphe_2_ and Nspe_2_, the Cartesian coordinates of the 17 peptide backbone heavy atoms were extracted. The Euclidean distance matrix upper triangle was computed for these 17 shared backbone atoms, resulting in a 136-dimensional vector representation for each trajectory frame. These vector representations, concatenated across all sequences and holding stages and each interatomic distance, were standardized with Z-score normalization. Finally, the first two principal components were calculated with PCA-whitening using the scikit-learn python package.^42^ PCA of Nphe_3_ and Nspe_3_ was performed in a similar manner with the exception that the shared molecular subgraph of these were composed of 21 backbone heavy atoms.

### Nonequilibrium Green’s Function-Density Functional Theory (NEGF-DFT)

Representative molecular conformations generated of the most probable conformations by MD were used in quantum mechanics (QM) calculations to enable direct comparison between theory and experimental results. Nonequilibrium Green’s function-density functional theory (NEGF-DFT) calculations for Nphe_2_, Nspe_2_, Nphe_3_, and Nspe_3_ were performed with double-ζ (DZ) basis sets for gold atoms and double-ζ polarized (DZP) basis sets for carbon, hydrogen, oxygen, sulfur, and nitrogen. NEGF-DFT calculations were performed with a DFT-based nonequilibrium Green’s function (NEGF) approach using the TranSiesta and Tbtrans package.^43–45^ Electrode configurations contain 8 layers of 16 gold atoms along with a pyramid of 9 Au atoms. Sulfur atoms in the peptoids were made to interact with the gold atoms using a trimer binding motif, as previously described.^11^ Geometry relaxation of the sequences were performed using generalized gradient approximation-Perdew–Burke–Ernzerhof (GGA-PBE) functional^46^ using the TranSiesta package.^43^ DZ basis sets were used for all the gold atoms. DZP basis sets were used for carbon, hydrogen, oxygen, sulfur, and nitrogen. Electrode calculations were carried out with a 4 × 4 × 50 *k*-mesh. The geometry relaxation was carried out using a 4 × 4 × 1 *k*-mesh, which was performed until all forces were <0.05 eV/Å. After the junction was relaxed, the transport calculations were carried out using the TranSiesta package with the same functionals, basis sets, pseudopotential, and *k*-mesh as the geometry relaxation.^44,45^ Convergence was assessed prior to transmission calculations, using a real axis integration internal from −40 eV to infinity;^44^ this includes a crossing in the imaginary axis at 2.5 eV, and the γ value is −10*k*_B_ *T*. The circle grid consists of 102 G-Legendre points, and 15 G-Fermi points for the tail portion. Tbtrans was used to carry out the NEGF calculations and to obtain electron transmission as a function of energy (relative to the Fermi energy level).^45^ NEGF calculations were carried out from −3 to 3 eV with 0.05 eV energy increments.

## Results and Discussion

A library of peptoid oligomers (*n* = 4, 5) with different side chains was prepared for molecular electronics experiments (Figure 1a). Peptoids were designed to contain thiomethyl (-SCH_3_) monomers at the termini to readily bind to gold, thereby providing electrical contacts to metal electrodes in STM-BJ (Figure 1b).^47^ Aside from terminal thiomethyl anchors, peptoids are chemically complex molecules containing a C-terminal amide and tertiary and secondary amines. C-terminal amides feature a lone pair of electrons on the nitrogen atom that are delocalized through resonance with the carbonyl group, significantly reducing their ability to serve as anchors. Peptoids also contain sterically constrained tertiary amides which are unlikely to interact with gold electrodes and an internal secondary amine. Secondary amines have been previously shown to serve as weak anchor groups in molecular break-junction experiments,^48,49^ but such contacts typically give rise to continuously decaying molecular conductance features^48,49^ rather than the quasi-plateau-like conductance features observed for peptoids in this work (Figure 1c). To further assess the possibility of multiple anchoring motifs due to the secondary amine, we performed unsupervised machine-learning using silhouette-score clustering and Gaussian mixture modeling (Supporting Information Section S3). In all cases, results from cluster analysis are consistent with the absence of multiple anchoring motifs for these molecules.

**Figure 1:**
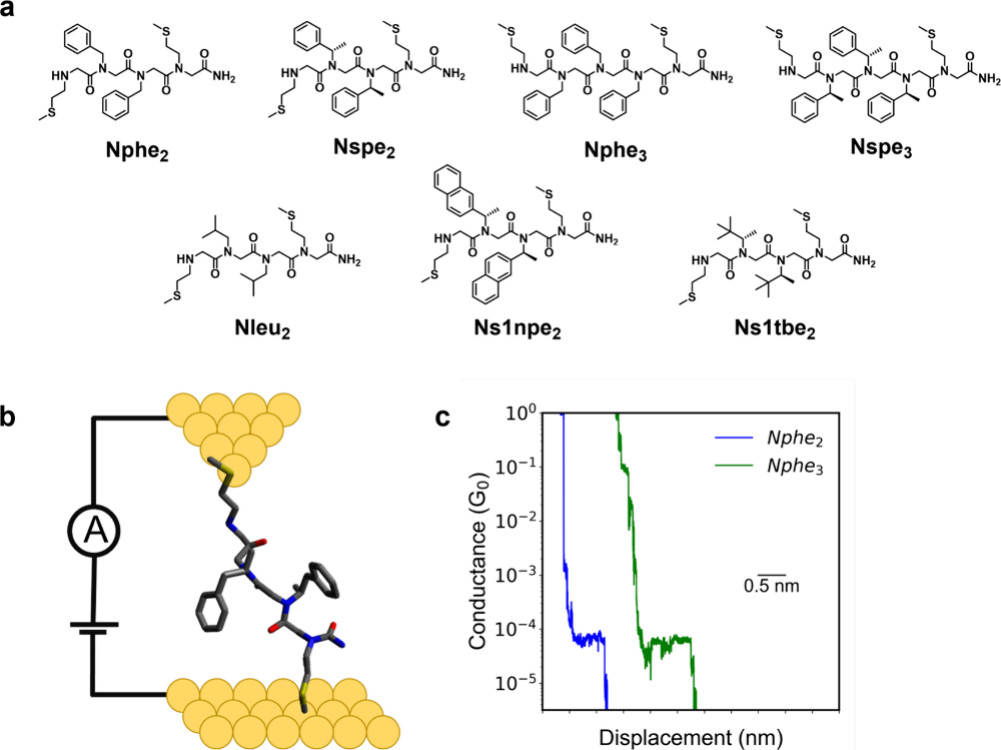
Overview of single-molecule electronic characterization of peptoids. (a) Chemical structures of peptoid oligomers classified by aromaticity and helicity. (b) Schematic of a molecular junction containing peptoid oligomer Nphe_2_. (c) Characteristic single-molecule conductance traces for Nphe_2_ and Nphe_3_ from STM-BJ experiments.

Peptoids were designed with chemically diverse monomers, including both aromatic and nonaromatic side chains, to understand the role of delocalized π-electrons in the side chains on conductance. We also selected monomers known to promote different degrees of backbone conformational constraints to understand how local backbone organization influences electron transport. The introduction of a methyl group at the N–C_α_ position on the monomer side chain induces steric constraints that bias the backbone toward locally ordered conformations compatible with a helical fold (Figure 1a).^50^ The Nspe monomer is a well-known, helix-inducing peptoid monomer for longer peptoid backbones, whereas Nphe lacks an N–C_α_ methyl group but is otherwise structurally identical to Nspe. In this way, precise molecular design and selection of peptoid monomers allow for investigation of how backbone conformation affects molecular electron transport. CD measurements show that Nspe_2_ and Nspe_3_ exhibit weak spectral features at approximately 190, 202, and 220 nm in water, whereas Nphe_2_ and Nphe_3_ do not show comparable spectral signatures (Figure S15). These features are interpreted as qualitative indicators of increased local conformational constraints, rather than evidence of a fully developed helical secondary structure, particularly given the relatively short oligomer chain lengths studied in this work.^51,52^ We further selected peptoid monomers Ns1npe and Ns1tbe which are known to promote backbone conformational constraints resulting in helical structures in longer peptoid oligomers.^53,54^ Finally, we included the monomer Nleu as a control that is neither aromatic nor associated with significant conformational constraints.

### Single-Molecule Conductance Measurements (STM-BJ)

We began by characterizing peptoids with aromatic monomers (Nphe_2_ and Nphe_3_) containing a benzyl group as the side chain but lacking substitution at the N–Cα position. Results from STM-BJ experiments reveal the presence of a well-defined conductance population, as observed by the plateau features in characteristic single-molecule conductance traces (Figure 1c). 1D and 2D molecular conductance histograms (Figures 2a-**d**) determined across large molecular ensembles indicate the presence of a conductance population around ∼10^–4.5^ *G*/*G*_0_ for Nphe_2_ and Nphe_3_, where *G*_0_ is the quantum unit of conductance. Based on these results, we posited that peptoid sequences lacking substitution at the N–C_α_ position (such as Nphe_2_ and Nphe_3_) give rise to a well-defined conductance population, characterized by a clearly defined mean and relatively low variance across a large ensemble of approximately 5,000–10,000 molecular traces.

**Figure 2:**
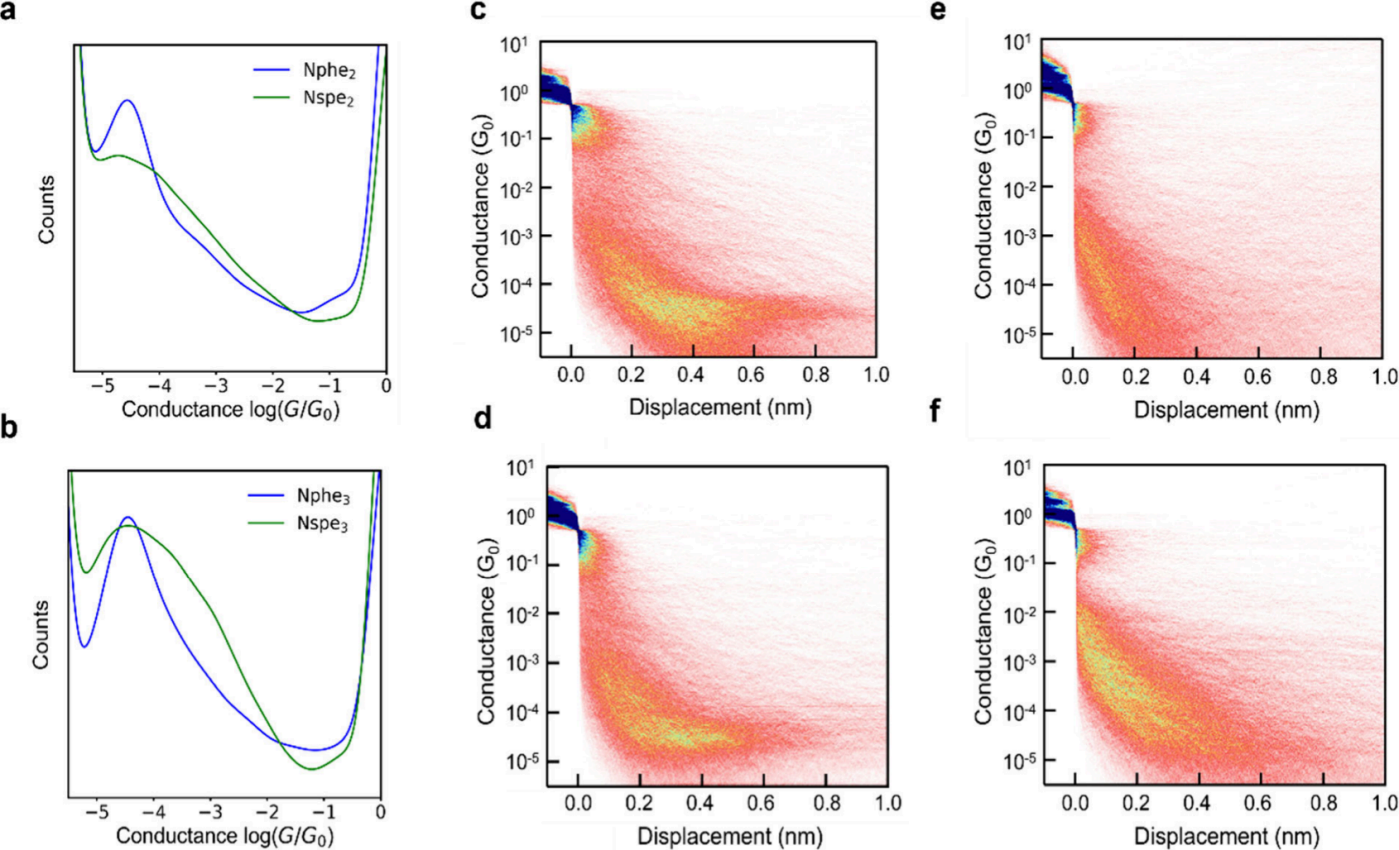
Single-molecule electronic measurements of peptoid oligomers at 250 mV applied bias in water. (a) 1D conductance histograms of Nphe_2_ (blue) and Nspe_2_ (green). (b) 1D conductance histograms of Nphe_3_ (blue) and Nspe_3_ (green). (c) 2D conductance histogram of Nphe_2_. (d) 2D conductance histogram of Nphe_3_. (e) 2D conductance histogram of Nspe_2_. (f) 2D conductance histogram of Nspe_3_. The 2D conductance histograms are color-coded by trace density, with darker blue colors indicating higher counts (higher probability of occurrence) and lighter colors indicating lower counts. All data were obtained using 0.1 mM concentrations of peptoids in water at 250 mV applied bias across ensembles of at least 5000 single molecules.

To understand how substitutions at the N–C_α_ position affect electron transport, we performed STM-BJ experiments on peptoid oligomers Nspe_2_ and Nspe_3_ containing a methyl group at the N–C_α_ position. Our results show that Nspe_2_ and Nspe_3_ exhibit a conductance population at ∼10^–4.5^ *G*/*G*_0_ (Figures 2e,f). However, it should be noted that the conductance populations for Nspe_2_ and Nspe_3_ are broader and generally occur over a shorter junction distance range compared to Nphe_2_ and Nphe_3_. We further characterized the electron transport behavior for control molecules Ns1tbe_2_ and Ns1npe_2_, which contain a methyl group at the N–C_α_ position but differ in the side group compared to Nspe_2_ and Nspe_3_. Whereas Nspe_2_ and Nspe_3_ contain a benzyl group in the side chain, Ns1tbe_2_ and Ns1npe_2_ contain a *t*-butyl group and a naphthyl group in their side chains, respectively, in addition to the methyl group at the N–C_α_ position. Our results show that control molecules Ns1tbe_2_ and Ns1npe_2_ similarly lack the well-defined conductance feature observed in Nphe_2_ and Nphe_3_ (Figures S18, S19). Overall, these experiments suggest that the introduction of a methyl group at the N–C_α_ position for several different side group chemistries disrupts the low conductance feature observed for Nphe_2_ and Nphe_3_ in 1D conductance histograms.

To further understand the role of side group chemistry on electron transport in peptoids, we performed STM-BJ experiments on the peptoid oligomer Nleu_2_. Similar to Nphe_2_ and Nphe_3_, Nleu_2_ contains a hydrogen atom at the N–C_α_ position but lacks an aromatic ring on the side group. Our results show that the well-defined conductance feature is absent in Nleu_2_ (Figures S18, S20). The mean conductance value of Nleu_2_ (∼10^–4.8^ *G*/*G*_0_) is smaller than the mean conductance values of Nphe_2_ and Nphe_3_ (Table S1), which suggests that the lack of an aromatic group in the side chains results in a slightly decreased conductance due to the lack of delocalized π-electronics in the side chains. Although all peptoids studied in this work exhibit a discernible conductance population in the one-dimensional (1D) conductance histograms (Figures 2, Figures S18–S20), clear differences are observed in the breadth of these distributions. To quantify these differences, we determined the full width at half-maximum (fwhm) of the 1D conductance histograms. Notably, Nphe_2_ and Nphe_3_ show narrower distributions with fwhm values of approximately 1.4, whereas all other peptoids exhibit broader distributions with fwhm values of approximately 1.8. These results indicate that Nphe_2_ and Nphe_3_ exhibit well-defined conductance populations characterized by a clearly defined mean and relatively low dispersion across a large molecular ensemble.

We further sought to understand the molecular origins of the differences in conductance behavior for Nphe_2_ and Nphe_3_ compared to related oligomers. The absence of the N–C_α_ methyl group (in Nphe_2_ and Nphe_3_) reduces conformational constraints and increases the likelihood of the development of a *trans* conformation that promotes electron transport across the junction.^50,51^ It is well established that substitution at the N–Cα position favors *cis* peptoid conformations over *trans* conformations, thereby introducing increased steric constraints in the backbone.^50,51^ Although methyl substitution at the N–C_α_ position is known to increase local conformational constraints and influence cis–trans amide preferences, our results indicate that these constraints lead to increased heterogeneity in accessible junction conformations under STM-BJ conditions, resulting in broader conductance distributions rather than a narrower, converged conductance population. Overall, our results indicate that peptoid sequences with aromatic side groups and without chemical substitutions at the N–C_α_ position give rise to defined low conductance features.

Prior work has demonstrated that peptides of similar length as studied in this work show a conformation-dependent electron transport behavior due to intramolecular H-bonding that results in a bimodal conductance distribution,^16^ which is markedly different from the unimodal conductance populations observed for peptoids. Furthermore, alkane chains of similar contour length (1,16-hexanedithiol) compared to the peptoid oligomers in the work do not exhibit a well-defined molecular conductance feature.^16,55–58^ From this view, the single-molecule conductance measurements suggest that peptoids containing aromatic side groups without substitution at the N–C_α_ position exhibit a defined conductance feature, which is markedly different than the conductance behavior in analogous molecules with nonconjugated backbones such as peptides and alkanes. Moreover, our single-molecule electronic measurements of peptoids reveal a slightly larger mean conductance value for Nphe_3_ compared to Nphe_2_ or Nspe_2_ (Table S1). These results suggest that factors such as molecular conformation can influence electron tunneling pathways, such that molecular junction displacement is not the sole determining factor for electron tunneling currents.^59,60^

### Molecular Dynamics (MD) Simulations

We performed all-atom MD simulations for peptoid oligomers to understand the role of molecular conformation on electron transport (Figure 3a), following the methodology established in prior work^16^ (Figures S21–23). Our results show that peptoids primarily adopt orthogonal binding conformations, such that the vector between S atoms on terminal anchor groups is highly aligned with the molecular pulling direction (Figures S22–S23), denoted by the presence of a single dominant free energy basin. The peptoid oligomers studied in this work are chemically complex with several potential H-bonding sites. We began by assessing the role (if any) of putative intramolecular H-bonding along peptoid backbones. Nphe_2_ and Nspe_2_ contain two potential H-bond donors and four potential H-bond acceptors, whereas Nphe_3_ and Nspe_3_ potentially contain two H-bond donors and five acceptors (Figure 3a). Hydrogen bonding analysis was performed from MD simulations based on all possible donor–acceptor combinations between backbone nitrogen atoms and backbone carbonyl oxygen atoms at various interanchor displacement potentials of 6 Å, 9 Å, and 12 Å holding stages (Figure 3a–c, and Figures S24, S25) and our results indicate the lack of prominent hydrogen bonding interactions. We further examined CH–O interactions between backbone methylene groups and carbonyl oxygens and did not observe prominent H-bonding interactions (Figures S26–S29). This analysis reveals that there are no significant intramolecular H-bonds for any of the peptoids studied in this work. The results differ significantly from peptide backbones where a distinct bimodal population is observed in the conformational space in MD simulations and in conductance populations from STM-BJ experiments.^16^ Overall, these results indicate that H-bonding does not significantly influence electron transport pathways for peptoids.

**Figure 3:**
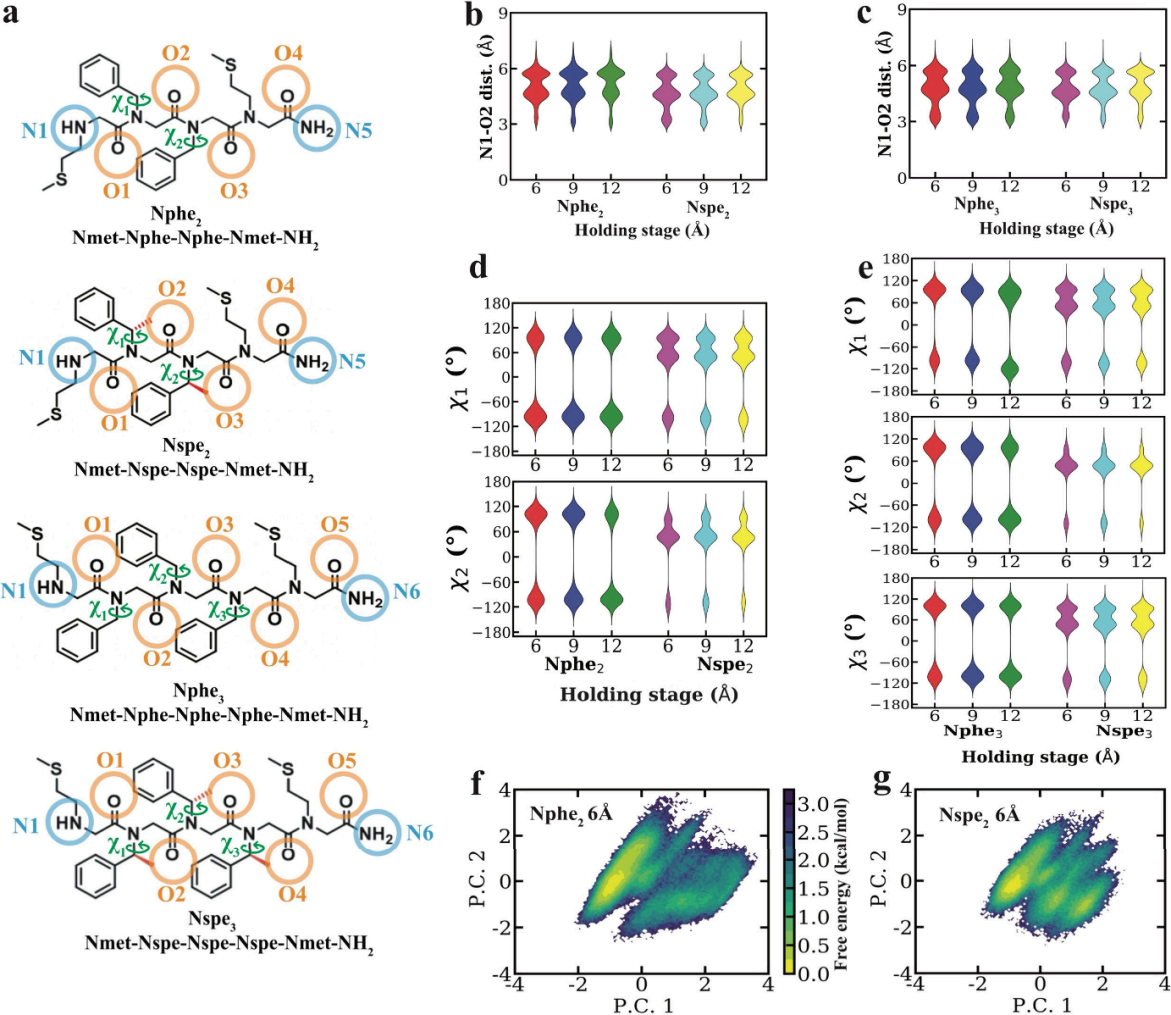
All-atom molecular dynamics (MD) simulations for peptoid oligomers. (a) Schematic representation of angles and atoms used for dihedral angle analysis for Nphe_2_, Nspe_2_, Nphe_3_, and Nspe_3_. Distribution of potential hydrogen bonding distances between N1 and O2 for (b) Nphe_2_ and Nspe_2_ at 6 Å, 9 Å, and 12 Å holding distances and (c) Nphe_3_ and Nspe_3_ at 6 Å, 9 Å, and 12 Å holding distances. (d) Distribution of dihedral angles χ_1_ and χ_2_ around the nitrogen and N–C_α_ bonds for Nphe_2_ and Nspe_2_ at 6 Å, 9 Å, and 12 Å holding distances. (e) Distribution of dihedral angles χ_1_, χ_2_ and χ_3_ around the nitrogen and N–C_α_ bonds for Nphe_3_ and Nspe_3_ at 6 Å, 9 Å, and 12 Å holding distances. (f) PCA analysis for Nphe_2_ at 6 Å holding distance. (g) PCA analysis for Nspe_2_ at 6Å holding distance.

To compare the electron transport behavior between different peptoid backbones, we analyzed the dihedral angle χ_*i*_ around the nitrogen and N–C_α_ bond to understand how steric interactions influence electron transport (Figure 3a). Nphe_2_ and Nphe_3_ show two dominant populations at large dihedral angles of ± 100° whereas Nspe_2_ and Nspe_3_ show three populations, with a smaller population at ± 100°, and most of their population lying between 0° and 80° (Figure 3d,e). We emphasize that these results do not necessarily imply increased equilibrium conformational diversity upon N–C_α_ substitution. Rather, consistent with prior work,^50,51^ N–C_α_ substitution introduces steric constraints and biases the backbone toward cis-like conformations. We posit that the lack of conformational flexibility upon N–C_α_ substitution inhibits a molecule’s ability to adopt conformations or associations with the electrode for enhanced transport. In the present work, short peptoids are examined under nonequilibrium STM-BJ conditions, where a moderate applied bias (250 mV) across an approximately 1 nm electrode gap generates a strong local electric field (∼10^8^ V/m), which is explicitly incorporated into the MD sampling strategy. Results from backbone dihedral analysis, Ramachandran plots (Figures S30, S31), principal component analysis (Figure 3f,g and Figures S32, S33), and circular dichroism measurements (Figure S15) indicate that Nspe_2_ and Nspe_3_ possess more rigid backbones than Nphe_2_ and Nphe_3_. From this view, we attribute the well-defined conductance features observed in Nphe_2_ and Nphe_3_ to the increased backbone flexibility of these peptoid oligomers, together with a higher probability of adopting trans conformations in sequences containing Nphe monomers compared to those containing Nspe monomers. The absence of the N–C_α_ methyl group in Nphe_2_ and Nphe_3_ reduces conformational constraints and increases the likelihood of an extended backbone conformation that promotes electron transport across the junction. These results are consistent with recent work on the molecular electronics of amide-containing organic foldamers, where steric effects cause the foldamers to adopt distinct 3D conformations.^61^ In these systems, larger conductance is observed when the amide bonds are in a *trans* conformation rather than a *cis* conformation.^61^

### NEGF-DFT and Quantum Calculations

Representative molecular representations of the most probable conformations generated by MD were used in quantum mechanics (QM) calculations to enable direct comparison between theory and experimental results. Nonequilibrium Green’s function-density functional theory (NEGF-DFT) calculations for Nphe_2_, Nspe_2_, Nphe_3_, and Nspe_3_ were performed with double-ζ (DZ) basis sets for gold atoms and double-ζ polarized (DZP) basis sets for carbon, hydrogen, oxygen, sulfur, and nitrogen (Figure 4a). Results from transmission calculations show similar average electron transmission for Nphe_2_ and Nspe_2_ (Figure 4b), consistent with single-molecule experimental results. The dominant mechanism for nanoscale charge transport in small organic molecules has been reported as nonresonant coherent tunneling,^5,62–69^ in which conductance decays exponentially with molecular length. Whereas distance is a key factor in electron tunneling due to its exponential decay with length,^70^ additional factors such as molecular composition^27^ and molecular conformation^16^ significantly influence electron tunneling currents. Results from NEGF-DFT calculations are consistent with results from STM-BJ in showing slightly similar electron transmission for Nphe_3_ compared to Nphe_2_ and for Nspe_3_ compared to Nspe_2_ (Figure S34 and Table S2). Our single-molecule experimental results (Table S1) indicate comparable molecular conductance values for Nphe_3_ compared to Nphe_2_ and for Nspe_3_ compared to Nspe_2_, which is consistent with the notion that molecular junction distance is not the sole determinant of electron tunneling. Results from NEGF-DFT calculations (Table S2) further corroborate this claim as we observe similar electron transmission values for longer peptoids as compared to shorter peptoids.

**Figure 4:**
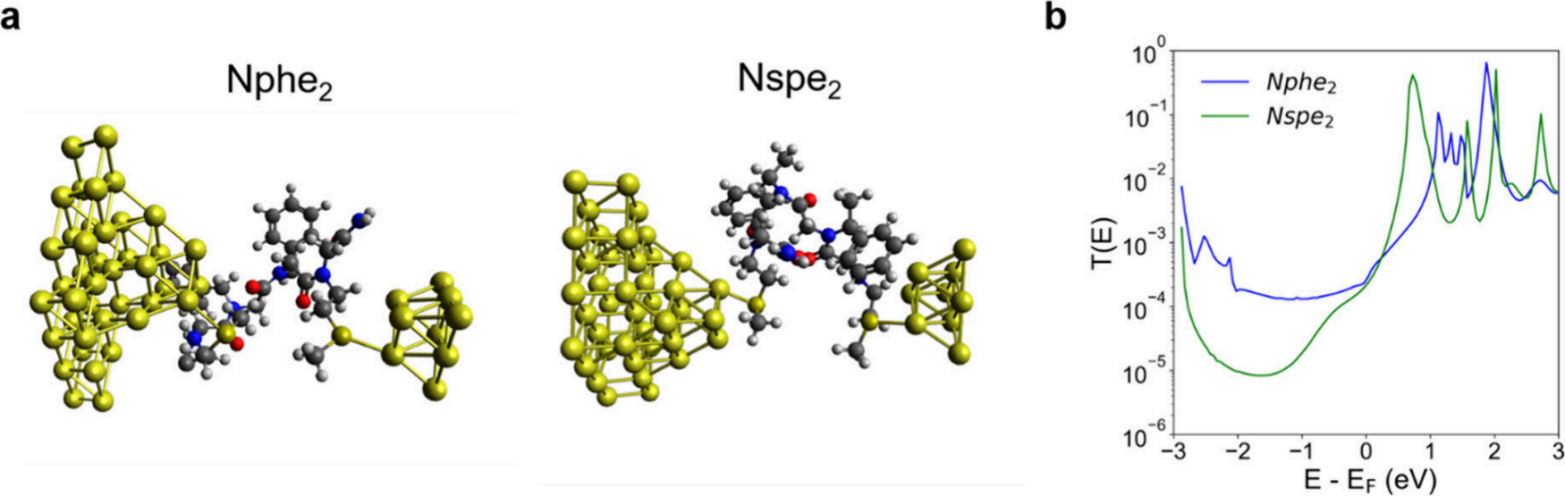
Nonequilibrium Green’s function-density functional theory (NEGF-DFT) calculations. (a) Junction schematics for Nphe_2_ and Nspe_2_. (b) Transmission plots for Nphe_2_ and Nspe_2_.

### Comparison between Peptides and Peptoids

To further understand the role of H-bonds on electron transport, we compared the molecular conductance behavior of peptoids to peptides. Molecular-scale experiments and modeling reveal distinct electron transport fingerprints for peptides and peptoids. Peptides exhibit dominant backbone H-bonding (Figure 5a), which gives rise to a bimodal conductance distribution (Figure 5b).^16^ This observation is supported by molecular dynamics (MD) simulations that identify a population below the H-bonding threshold, strongly suggesting that electron transport in peptides can occur through H-bond–mediated pathways (Figure 5c). On the contrary, peptoids lack backbone H-bonding interactions (Figure 5d), resulting in a unimodal conductance distribution that closely aligns with the low-conductance state observed for peptides (Figure 5e). The absence of H-bonded mediated electron transport pathways in peptoids is supported by all-atom molecular dynamics simulations (Figure 5f).

**Figure 5:**
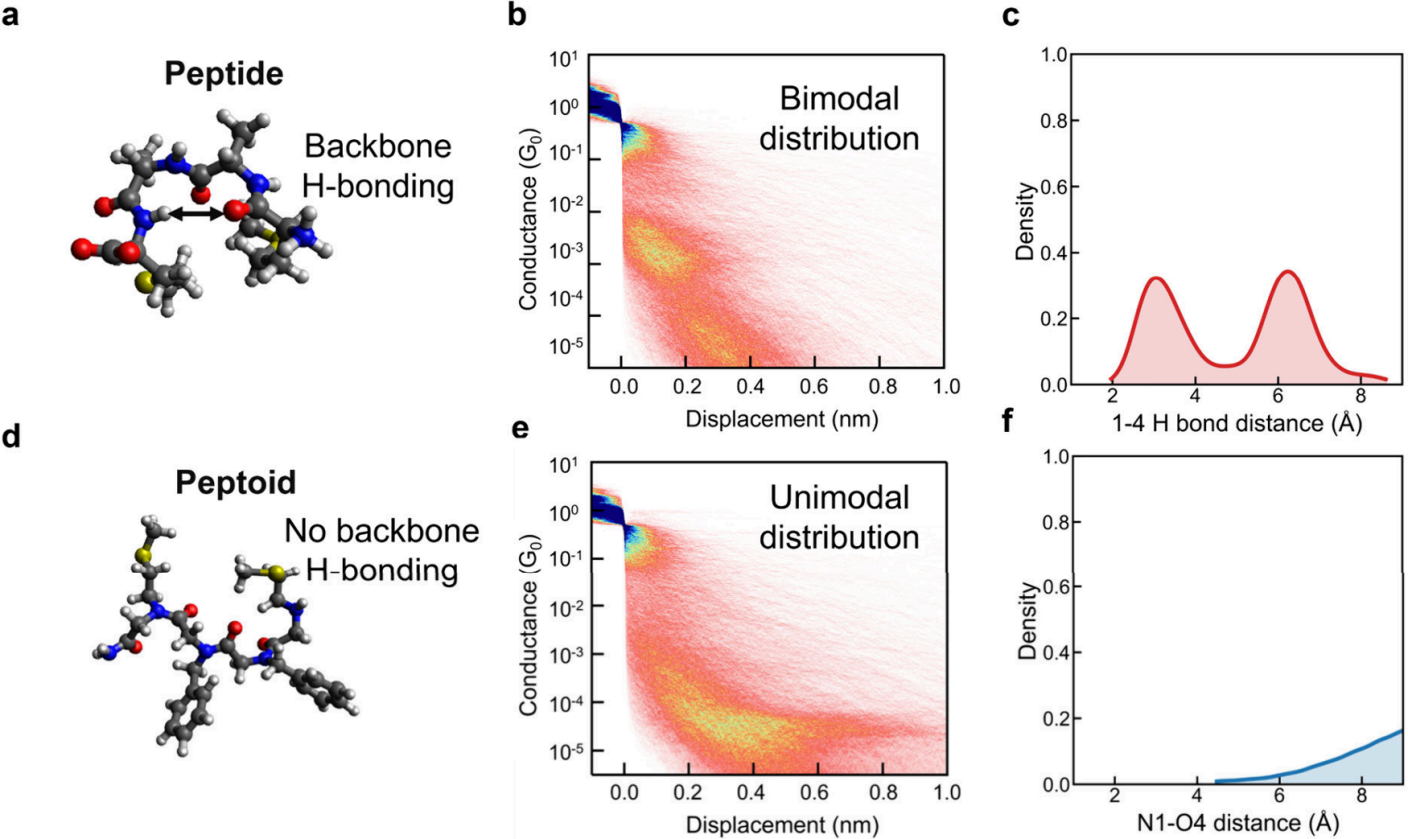
Comprehending differences in molecular-scale electronic fingerprints between peptides and peptoids. (a) Chemical structure of peptide MAAM, indicating the possibility of forming intermolecular hydrogen bonds. (b) Two-dimensional conductance histograms obtained from molecular-scale break junction experiments, showing a bimodal conductance distribution where the high-conductance state corresponds to a secondary-structure-mediated electron transport pathway. (c) All-atom molecular dynamics (MD) simulations illustrating the potential formation of hydrogen bonds in peptides, which can give rise to β-turn or 3_10_-helix conformations. (d) Chemical structure of peptoid Nphe_2_, indicating the absence of backbone hydrogen bonding. (e) Two-dimensional conductance histograms from molecular-scale break junction experiments showing a unimodal conductance distribution and the absence of a high-conductance state observed in peptides due to hydrogen bonding. (f) Results from all-atom MD simulations consistent with a lack of hydrogen-bond-mediated electron transport. 2D conductance histograms are color-coded by trace density, with darker blue colors indicating higher counts (higher probability of occurrence) and lighter colors indicating lower counts. All data were obtained using 0.1 mM concentrations of peptoids in water at 250 mV applied bias across ensembles of at least 5000 molecules.

We further compared the molecular-scale electronic fingerprints of peptides and peptoids with 1,16-hexadecanedithiol to contrast conductance decay between biomolecules and organic molecules of similar length.^16^ 1,16-Hexadecanedithiol contains a flexible alkane chain backbone and no possibility of intramolecular H-bonding. Our results show two conductance populations for peptides (around 10^–2.8^ *G*/*G*_0_ and 10^–4.2^ *G*/*G*_0_), one conductance population for peptoids (at ∼10^–4.5^ *G*/*G*_0_), but no significant conductance peaks are observed for the flexible alkane backbones, though a faint population is observed between ∼10^–1^–10^–2^ *G*/*G*_0_ arising due to the use of different anchors and strong coupling between the -SH terminal anchor groups and the gold electrode.^55^

To further investigate the role of H-bonding on electron transport, we used a bond counting methodology based on the tunneling pathway model.^71,72^ Generally, conductance decay is associated with through-bond, through-space, and through-H-bond electron transport with through-bond electron tunneling being the most efficient because of a lower potential barrier.^60,70^ Additionally, it can be assumed that the conductance decay through an H-bond is twice as large as the decay through a covalent bond. Figure S35 indicates that if transport were to occur entirely through-bond (in peptides or peptoids), then the transport pathway would be approximately three bonds longer with an order of magnitude smaller conductance compared to the case of combined through-bond and H-bond transport.^60^ Results from the bond counting methodology align closely with the experimental observed results for peptoids and with the differences observed for peptides and peptoids.

## Conclusions

In this work, the electronic properties of sequence-defined peptoids were characterized using a combination of chemical synthesis, single-molecule electronic experiments, all-atom MD simulations, and quantum mechanical calculations. Our results show that peptoid sequences with aromatic side groups and without substitutions at the N–C_α_ position (e.g., Nphe_2_ and Nphe_3_) give rise to a well-defined low conductance feature. Interestingly, the low-conductance features observed for Nphe_2_ and Nphe_3_ are absent in peptoid analogs containing a methyl group at the N–C_α_ position (Nspe_2_, Nspe_3_, Ns1tbe_2_, and Ns1npe_2_) or in peptoids lacking aromatic side chains (Nleu_2_). To rationalize these results, all-atom MD simulations are used to understand the conformational heterogeneity of peptoid backbones. Representative conformational states generated by MD simulations are used in quantum mechanical calculations, and the results are in reasonable qualitative agreement with the experiments. Our results show that the molecular-scale electronic fingerprint of peptoids is markedly different from that of peptides of similar length. At the single-molecule level, peptoids with aromatic side groups lacking H-bonds and without chemical substitutions at the N–C_α_ position exhibit well-defined conductance states. This behavior is fundamentally different than the single-molecule electronic properties of peptides, which are known to exhibit a high-conductance state due to H-bonding interactions. These findings highlight the role of H-bond mediated electron transport in biomolecules such as peptides. This work provides a foundation for rational design of peptoid-based electronic materials and motivates investigation of electron transport in peptoid oligomers at larger length scales.

## Supplementary Material



## Notes

Additional details for experimental and computational methods, including synthetic procedures, characterization data such as mass spectrometry and HPLC analysis, additional single-molecule conductance measurements, molecular dynamics simulations, and quantum mechanical and transmission function calculations. (PDF)

## ◇

Department of Chemical and Biological Engineering, Princeton University, 35 Ivy Lane, Princeton, NJ 08540
