# Supplementary material for: Single-Molecule Electron Transport in Peptoids
Source: J Phys Chem B. 2026 Mar 4;130(11):3054–64. doi: 10.1021/acs.jpcb.5c07788 (PMC13007016; doi:10.1021/acs.jpcb.5c07788)
Supplement: Supplementary file 1 [file jp5c07788_si_001.pdf]

Supplementary Information for:

Single-molecule electron transport in peptoids

Brittany Prempin[1,2†], Rajarshi Samajdar[2,3†], Hemani Chhabra[2], Moeen Meigooni[2,4], Aleksei Aksimentiev[2,4,5], Emad Tajkhorshid[1,2,4,6,7], Jeffrey S. Moore[1,2,8], Charles M. Schroeder[1,2,3,4,8‡*]

[1]Department of Chemistry, University of Illinois Urbana-Champaign, Urbana, Illinois, 61801
[2]Beckman Institute for Advanced Science and Technology, University of Illinois Urbana-Champaign, Urbana, Illinois, 61801
[3]Department of Chemical and Biomolecular Engineering, University of Illinois Urbana-Champaign, Urbana, Illinois, 61801
[4]Center for Biophysics and Quantitative Biology, University of Illinois Urbana-Champaign, Urbana, Illinois, 61801
[5]Department of Physics, University of Illinois Urbana-Champaign, Urbana, Illinois, 61801
[6]Department of Biochemistry, University of Illinois Urbana-Champaign, Urbana, Illinois, 61801
[7]Department of Bioengineering, University of Illinois Urbana-Champaign, Urbana, Illinois, 61801
[8]Department of Materials Science and Engineering, University of Illinois Urbana-Champaign, Urbana, Illinois, 61801

[†]Contributed equally, co-first author

*Corresponding author: Charles M. Schroeder, email: cschroeder@princeton.edu
[‡]Present address: Department of Chemical and Biological Engineering, Princeton University, 35 Ivy Lane, Princeton, NJ 08544

Table of Contents

S1. Chemical Synthesis and Characterization ………………………………………… S3

S2. Circular dichroism (CD) spectroscopy …………………………………………….. S11

S3. Gaussian mixture modeling (GMM) ……………………………………………….. S12

S4. Single-molecule electronic measurements ……………………………………….. S14

S5. Molecular dynamics (MD) simulations ……………………………………………. S16

S6. Non-equilibrium Green's function–density functional theory (NEGF-DFT) calculations ………………………………………………………………………….. S23

S7. Tunneling pathway model: Bond counting for pathway determination ………… S24

S8. References …………………………………………………………………… S26

S1. Chemical Synthesis and Characterization

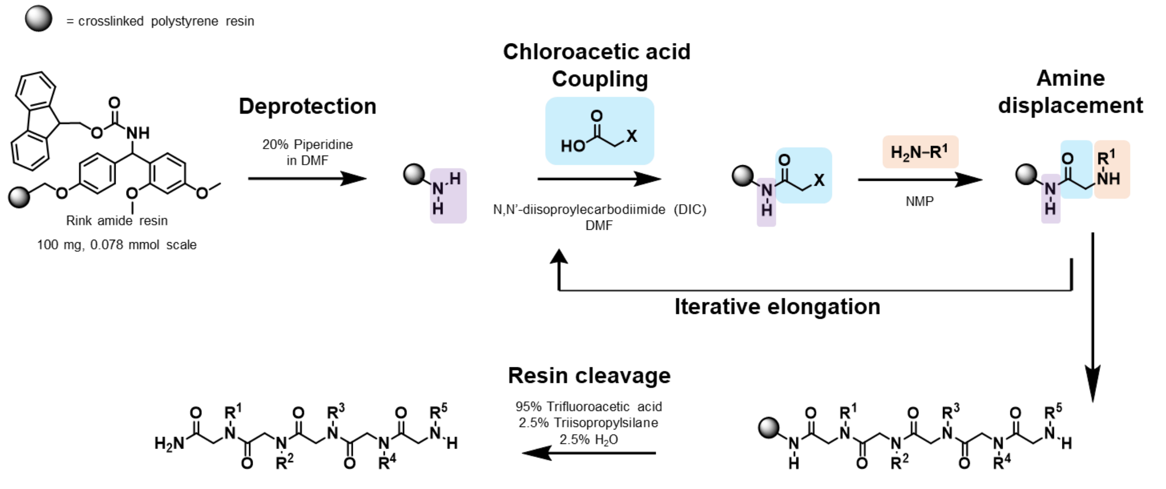

Scheme S1: Generalized scheme for the peptoid submonomer synthesis method.

Materials for peptoid synthesis

Rink amide resin (100-200 mesh, 0.78 mmol/g loading) was acquired from Novabiochem. *N*,*N*-dimethylformamide and methylene chloride (DCM) were purchased from Fischer Scientific. Piperidine was sourced from Aapptec. Chloroacetic acid, 2-(methylthio)ethylamine, (S)-1-phenylethylamine, benzylamine, (S)-1-(naphthalen-1-yl)ethan-1-amine, (S)-3,3-dimethylbutan-2-amine, triispropylsilane, and N-methylpyrrolidone were acquired from Sigma-Aldrich. Isobutylamine was bought from Ambeed. Diisopropycarbodiimide was sourced from Chem Impex. Trifluoroacetic acid was purchased from TCI. HPLC grade water was acquired from Honeywell.

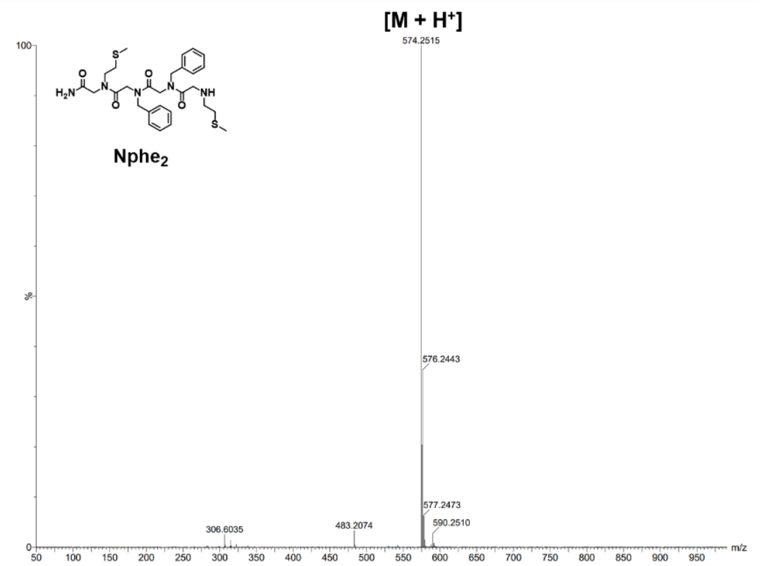

Fig. S1: HR ESI-MS characterization of $Nphe_2$, TOF detector, Positive mode. Calculated mass: 573.2443. Observed mass: 574.2515.

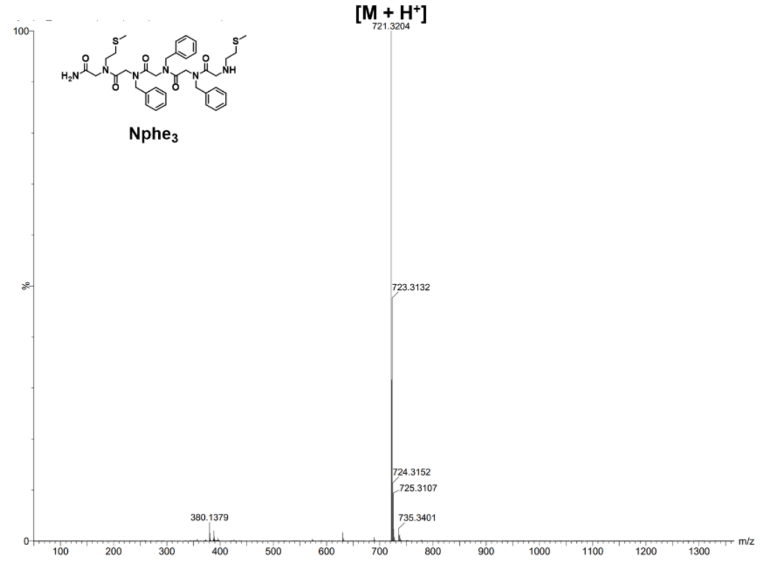

Fig. S2: HR ESI-MS characterization of $Nphe_3$, TOF detector, Positive mode. Calculated mass: 720.3128. Observed mass: 721.3204.

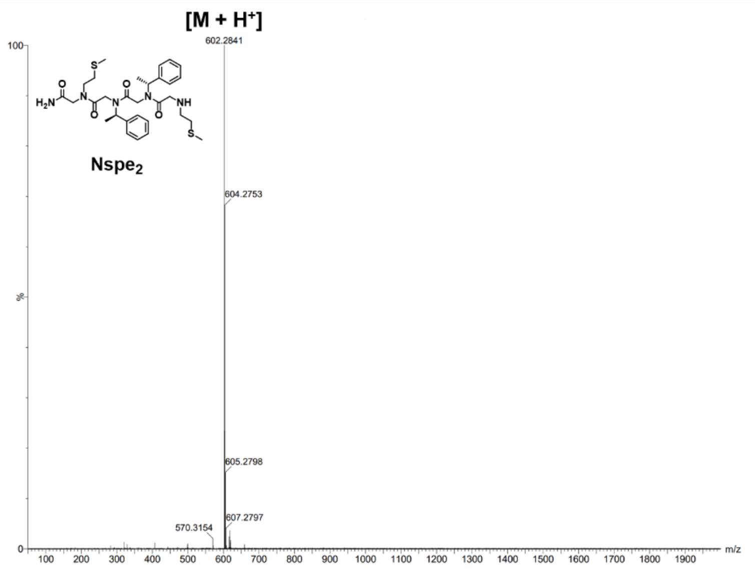

Fig. S3: HR ESI-MS characterization of Nspe$_2$, TOF detector, Positive mode. Calculated mass: 601.2756. Observed mass: 602.2841.

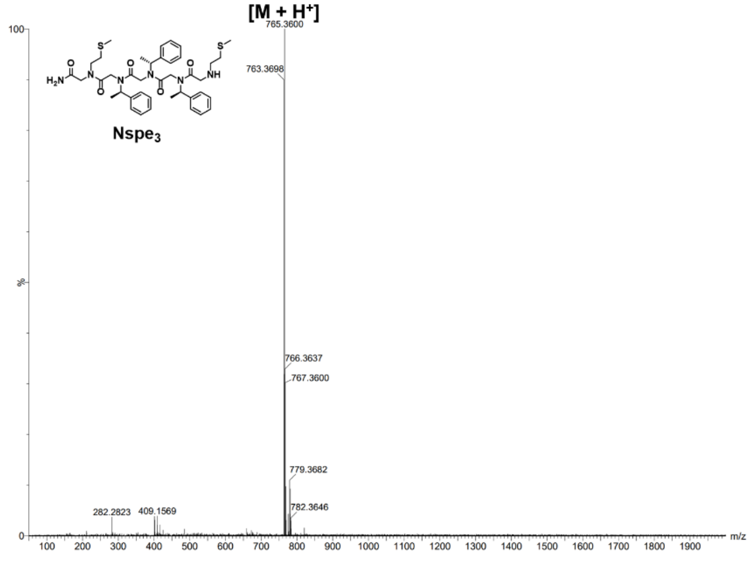

Fig. S4: HR ESI-MS characterization of Nspe$_3$, TOF detector, Positive mode. Calculated mass: 762.3597. Observed mass: 763.3698.

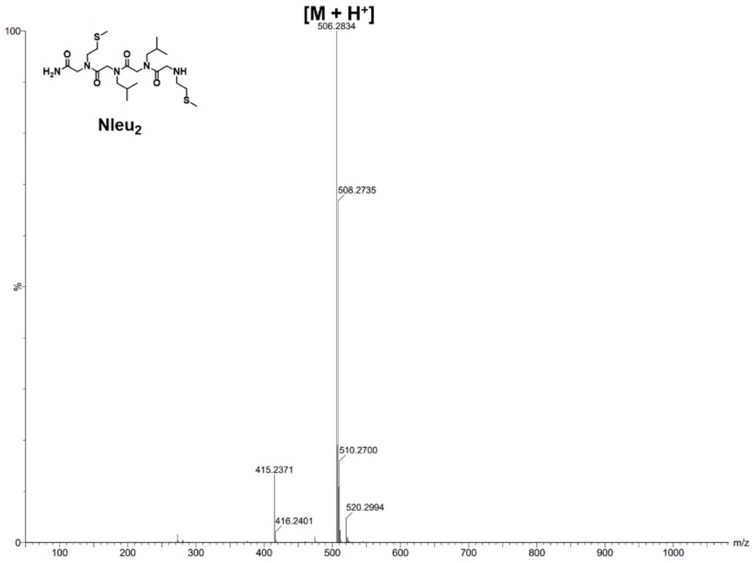

Fig. S5: HR ESI-MS characterization of Nleu$_2$, TOF detector, Positive mode. Caculated mass: 505.2756. Observed mass: 506.2834.

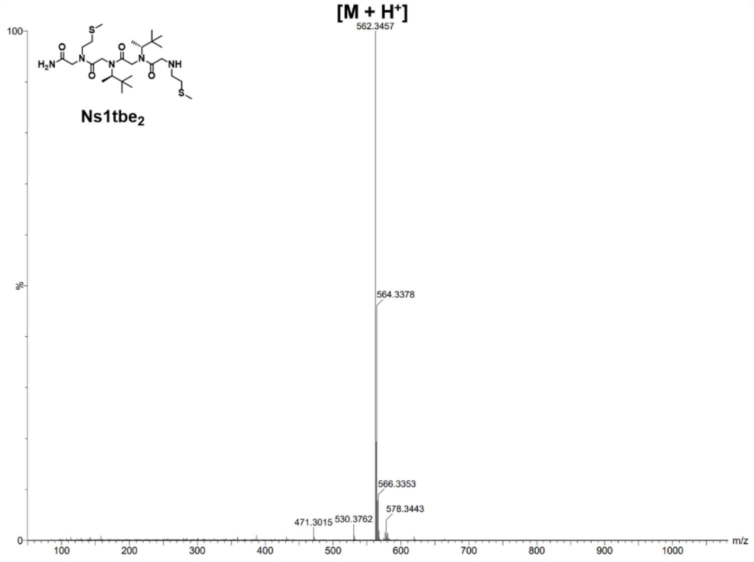

Fig. S6: HR ESI-MS characterization of Ns1tbe$_2$, TOF detector, positive mode. Calculated mass: 561.3382. Observed mass: 562.3457.

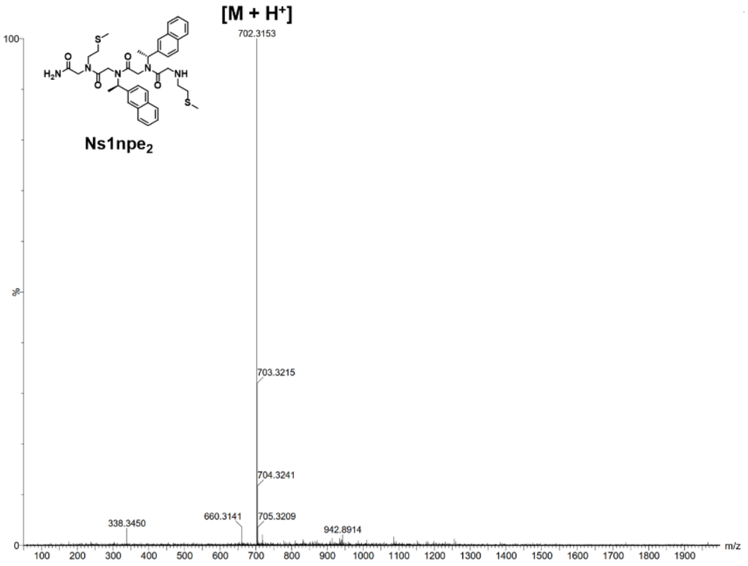

Fig. S7: HR ESI-MS characterization of Ns1npe$_2$, TOF detector, Positive mode. Calculated mass: 701.3069. Observed mass: 703.3153.

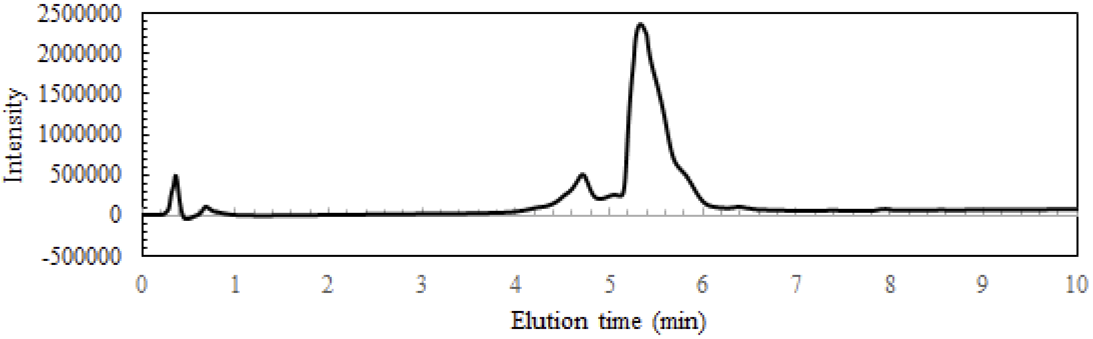

Fig. S8: Analytical HPLC trace for Nphe$_2$. Detection wavelength: 254 nm, 5% acetonitrile 95% water to 5% water 95% acetonitrile over 10 minutes, reverse phase C18 column. Calculated purity: 81.7%.

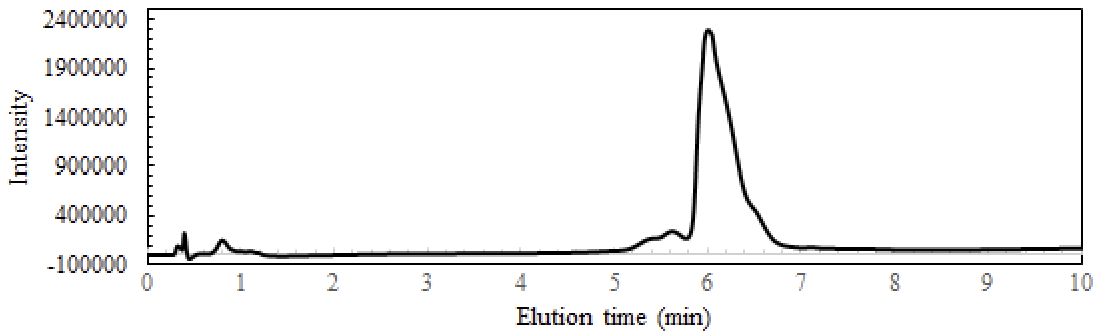

Fig. S9: Analytical HPLC trace for $Nphe_3$. Detection wavelength: 254 nm, 5% acetonitrile 95% water to 5% water 95% acetonitrile over 10 minutes, reverse phase C18 column. Calculated purity: 95.3%.

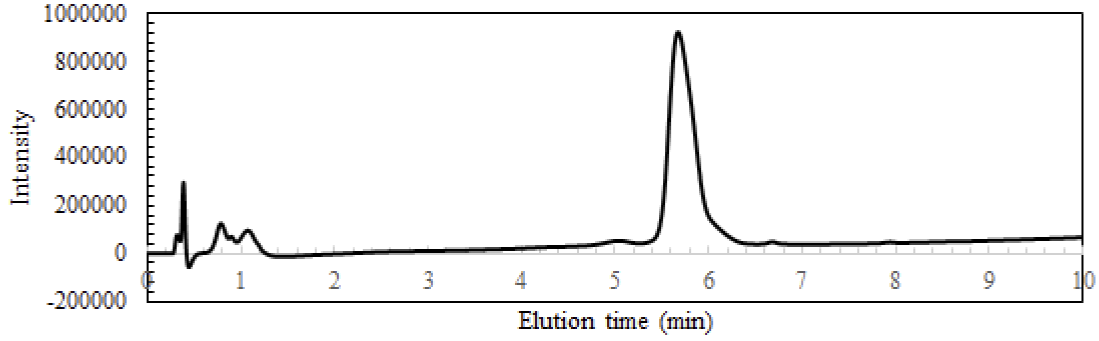

Fig. S10: Analytical HPLC trace for $Nspe_2$. Detection wavelength: 254 nm, 5% acetonitrile 95% water to 5% water 95% acetonitrile over 10 minutes, reverse phase C18 column. Calculated purity: 84.4%.

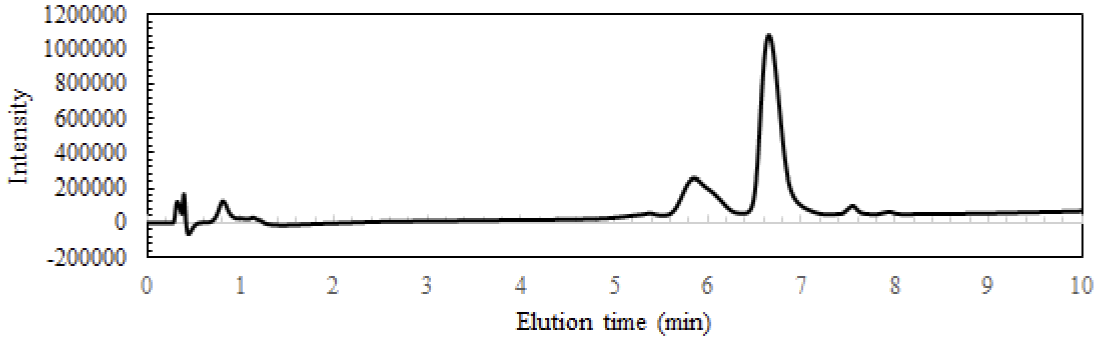

Fig. S11: Analytical HPLC trace for $Nspe_3$. Detection wavelength: 254 nm, 5% acetonitrile 95% water to 5% water 95% acetonitrile over 10 minutes, reverse phase C18 column. Calculated purity: 60.3%.

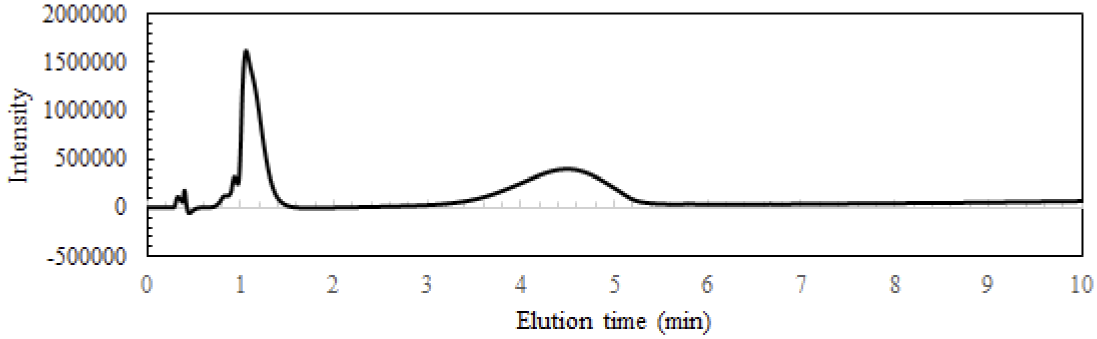

Fig. S12: Analytical HPLC trace for $Nleu_2$. Detection wavelength: 254 nm, 5% acetonitrile 95% water to 5% water 95% acetonitrile over 10 minutes, reverse phase C18 column. Calculated purity: 73.3%.

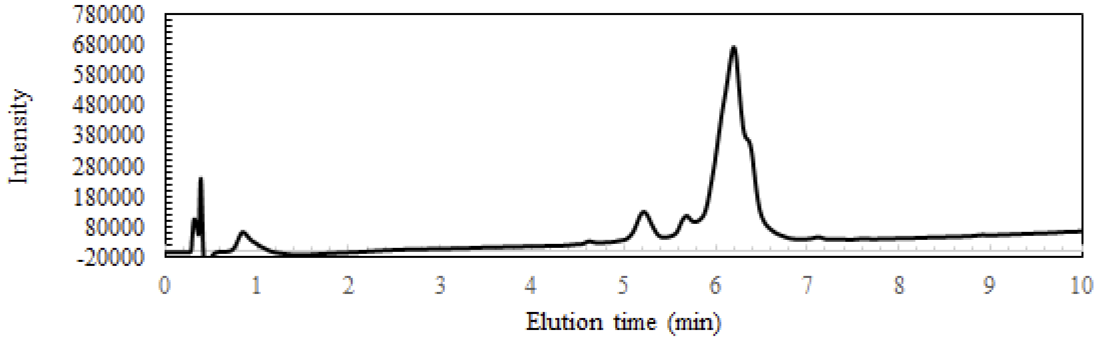

Fig. S13: Analytical HPLC trace for Ns1tbe$_2$. Detection wavelength: 254 nm, 5% acetonitrile 95% water to 5% water 95% acetonitrile over 10 minutes, reverse phase C18 column. Calculated purity: 99.9%.

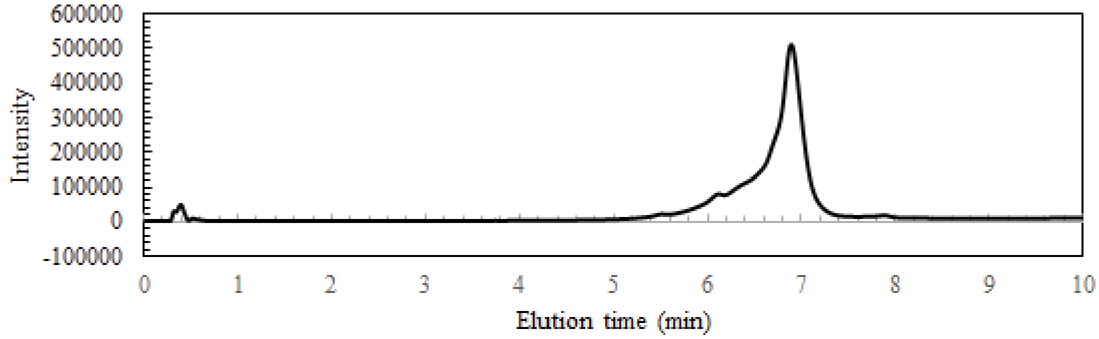

Fig. S14: Analytical HPLC trace for Ns1npe$_2$. Detection wavelength: 254 nm, 5% acetonitrile 95% water to 5% water 95% acetonitrile over 10 minutes, reverse phase C18 column. Calculated purity: 93.7%.

S2. Circular dichroism (CD) spectroscopy

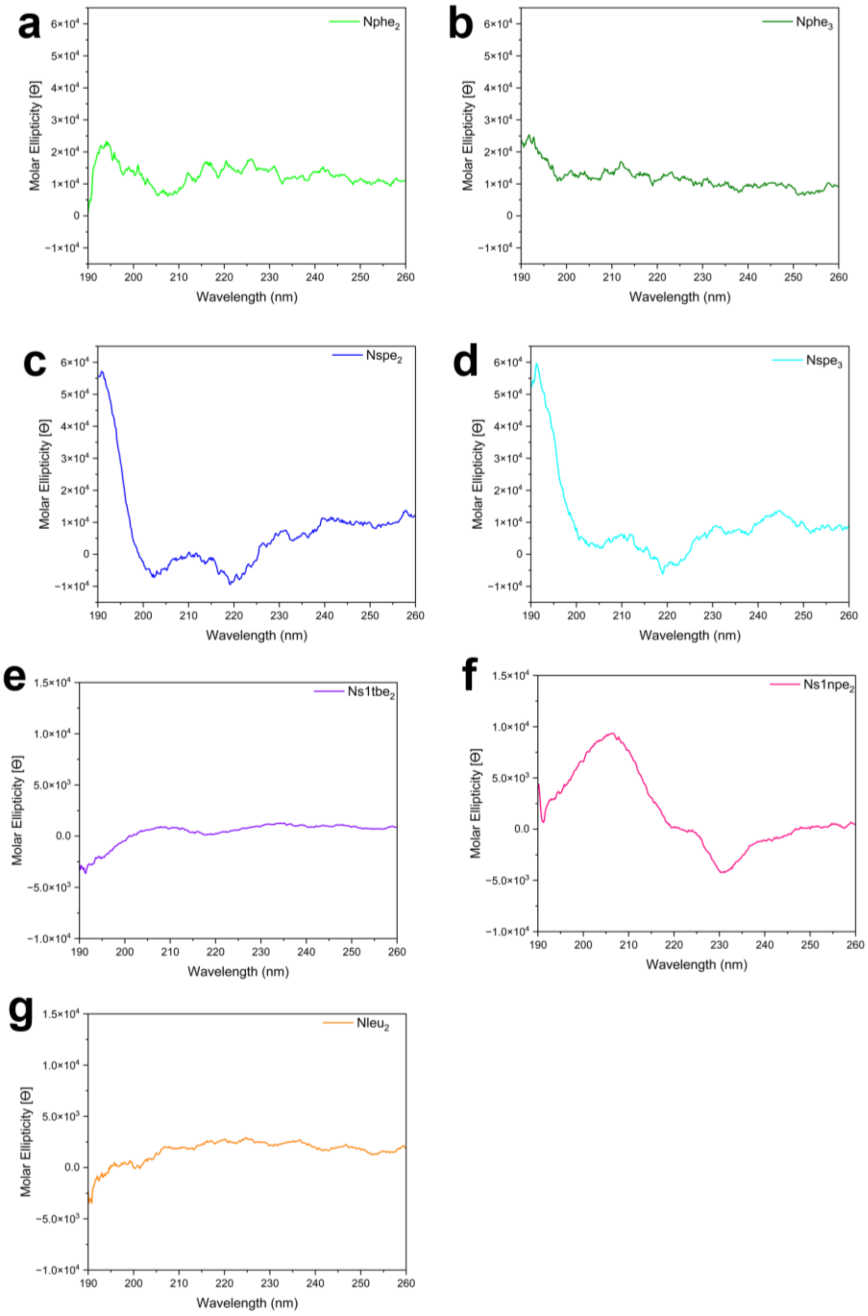

Fig. S15: Circular dichroism spectra for peptoids in this study. (a) $Nphe_2$, 3 µM in water (b) $Nphe_3$, 3 µM in water, (c) $Nspe_2$, 3 µM in water, (d) $Nspe_3$, 3 µM in water, (e) $Ns1tbe_2$, 40 µM in 50/50 water/acetonitrile, (f) $Ns1npe_2$, 20 µM in 50/50 water acetonitrile, (g) $Nleu_2$, 20 µM in 50/50 water/acetonitrile. Experiments were performed using spectral grade solvents at 25 °C, 10 scans per sample, solvent baseline removed. Measurements were taken on a Jasco J-1500 spectrophotometer.

S3. Gaussian mixture modeling (GMM)

To further assess the possibility of multiple electron-transport pathways arising from different anchoring motifs in peptoids, we applied unsupervised machine learning analysis using silhouette-score clustering and Gaussian mixture modeling (GMM), following a methodology established in prior work[1]. In the presence of multiple anchoring motifs arising from distinct anchor–electrode binding geometries, GMM would separate the conductance data into distinct clusters corresponding to different electron-transport pathways[16]. Our analysis indicates that the resulting silhouette scores are low (∼0.10) for two clusters and decrease further as the number of clusters increases. Results from GMM indicate that peptoids exhibit a single dominant electron transport pathway (**Figs. S16,S17**), indicating the absence of multiple anchoring motifs. Overall, unsupervised machine learning analysis using silhouette-score clustering and GMM illustrate that peptoids have electron transport behavior due to one anchor binding motif, which we posit to arise from the thiomethyl groups.

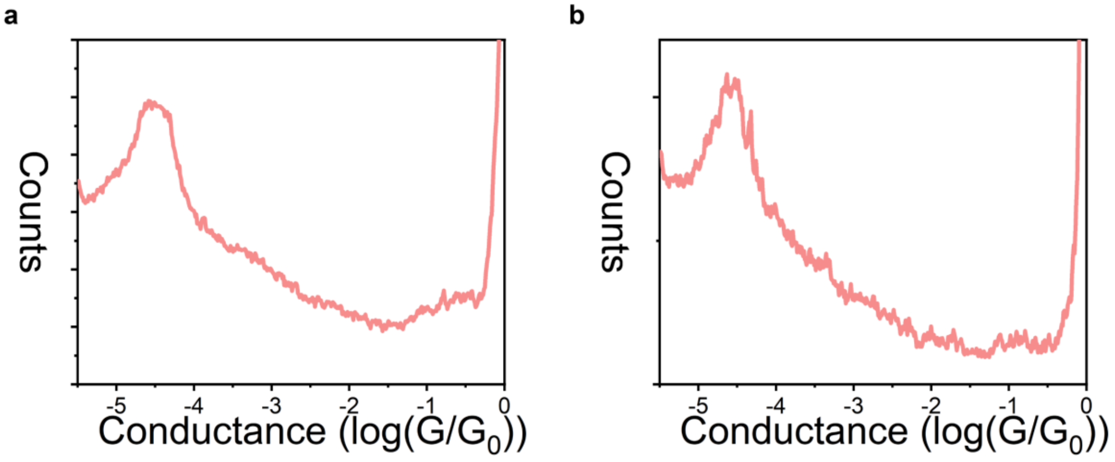

Fig. S16: Gaussian mixture modeling (GMM) for $Nphe_2$. Silhouette score clustering indicates that the optimal number of clusters is 2. (a) Cluster 1, which contains 80% of the data. (b) Cluster 2, which contains 20% of the data. GMM does not meaningfully separate the data into distinct clusters, which suggests the absence of multiple anchoring motifs during the break junction experiments.

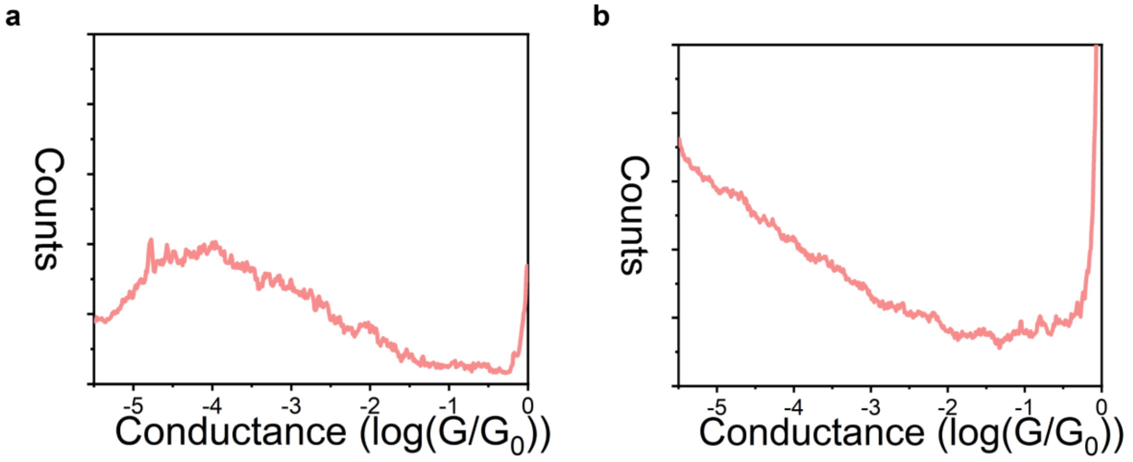

Fig. S17: Gaussian mixture modeling (GMM) for $Nspe_2$. Silhouette score clustering indicates that the optimal number of clusters is 2. (a) Cluster 1, which contains 87% of the data. (b) Cluster 2, which contains 13% of the data. GMM does not meaningfully separate the data into distinct clusters, which suggests the absence of multiple anchoring motifs during the break junction experiments.

S4. Single-molecule electronic measurements

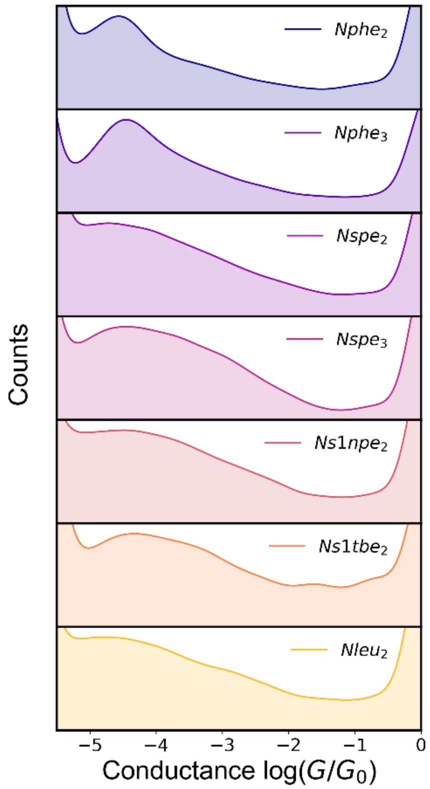

Fig. S18: 1D conductance histograms for all peptoids studied in this work.

Table S1: Conductance peak value and full width at half maximum for peptoid sequences studied in this work. The results are obtained for >5000 single molecules.

**Peptoid sequence**	**Conductance peak** [log(G/G_0)]
$Nphe_2$	-4.56
$Nphe_3$	-4.45
$Nspe_2$	-4.72
$Nspe_3$	-4.45
$Ns1tbe_2$	-4.30
$Ns1npe_2$	-4.50
$Nleu_2$	-4.80

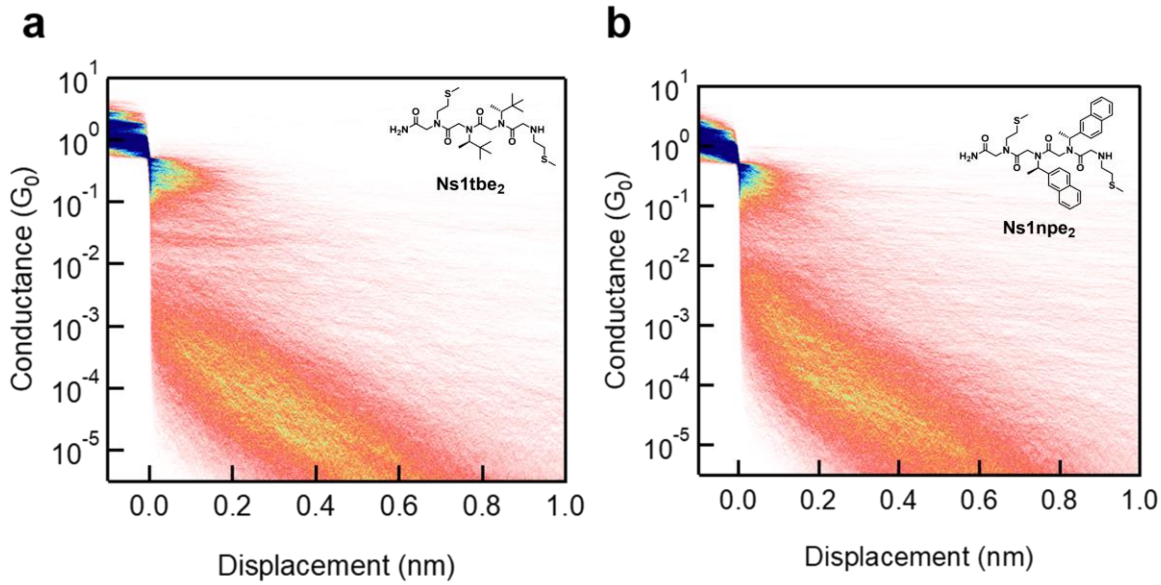

Fig. S19: 2D conductance histograms for (a) $Ns1tbe_2$, and (b) $Ns1npe_2$.

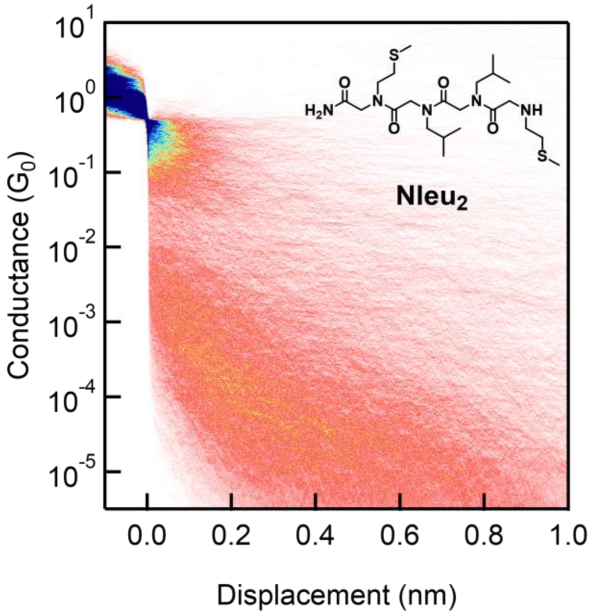

Fig. S20: 2D conductance histogram for $Nleu_2$.

S5. Molecular dynamics (MD) simulations

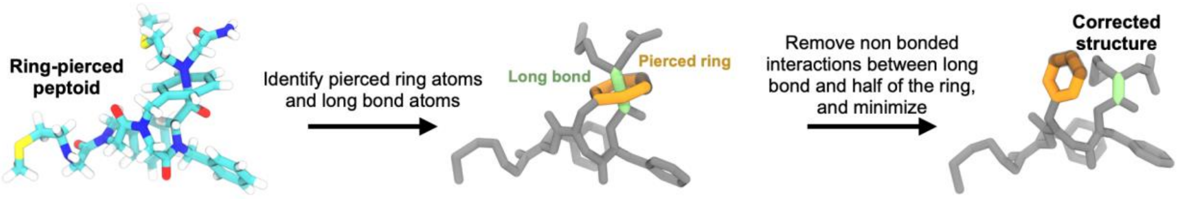

Fig. S21. Identifying and correcting ring piercings for molecular dynamics (MD) simulations based on peptoids. During backbone structure randomization and minimization, some peptoid bonds may randomly pierce a peptoid sidechain ring (left). Minimizing the structure results in and abnormally long bond that pierces the ring, which can be identified as any covalent bond with length greater than 2.5 Å, whereas the ring can be identified by comparing ring centers-of-mass with that of the atoms participating in the long bond (middle). Finally, nonbonded interactions between the long-bond atoms and half of the pierced ring (highlighted in grey) are removed and the structure is minimized, allowing the piercing bond to slip out of the ring, thus correcting the ring-pierced structure.

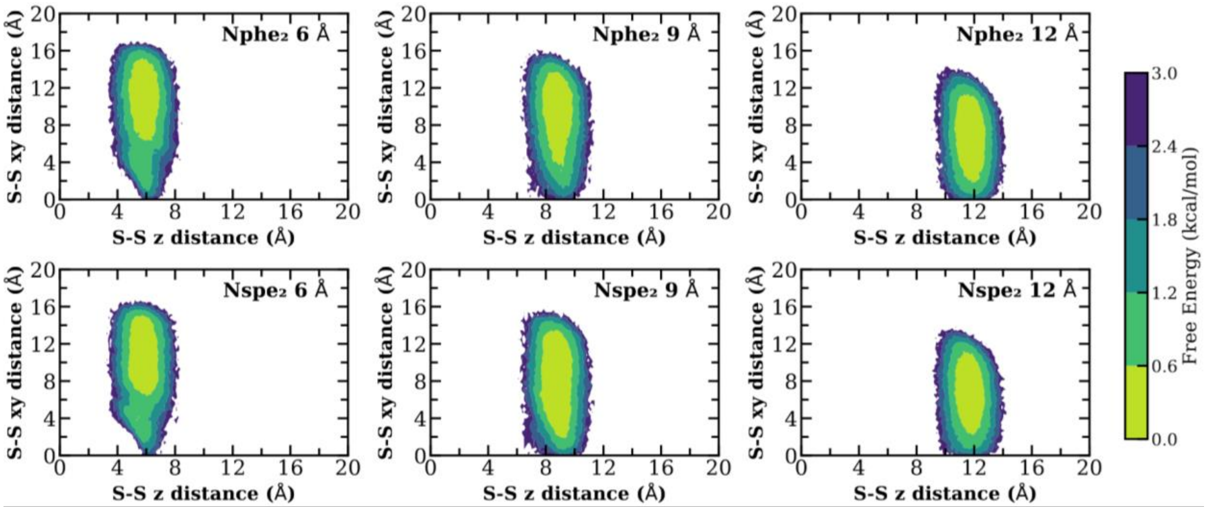

Fig. S22: Free energy plots for $Nphe_2$ and $Nspe_2$ at various inter anchor displacement potentials.

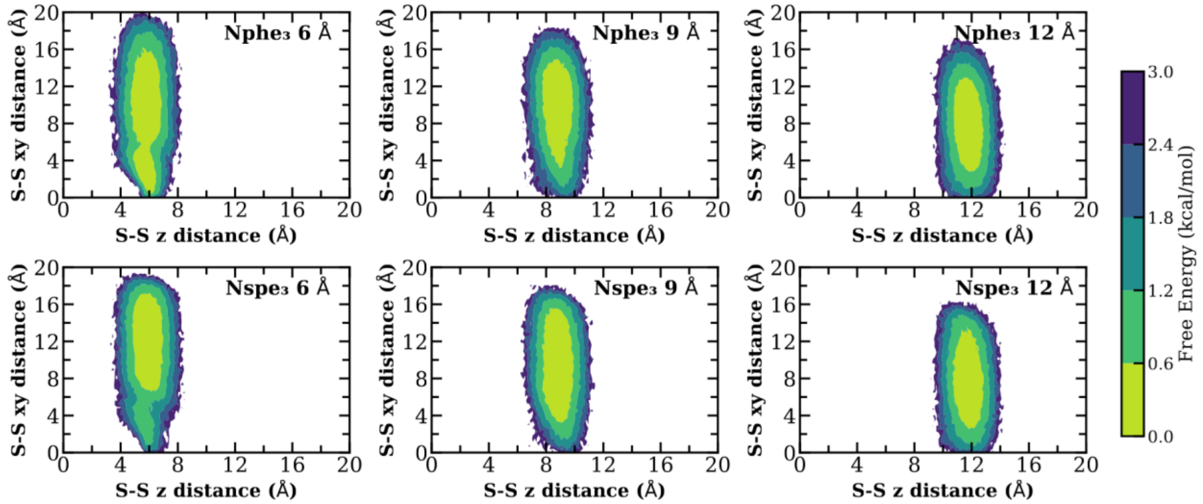

Fig. S23: Free energy plots for $Nphe_3$ and $Nspe_3$ at various inter anchor displacement potentials.

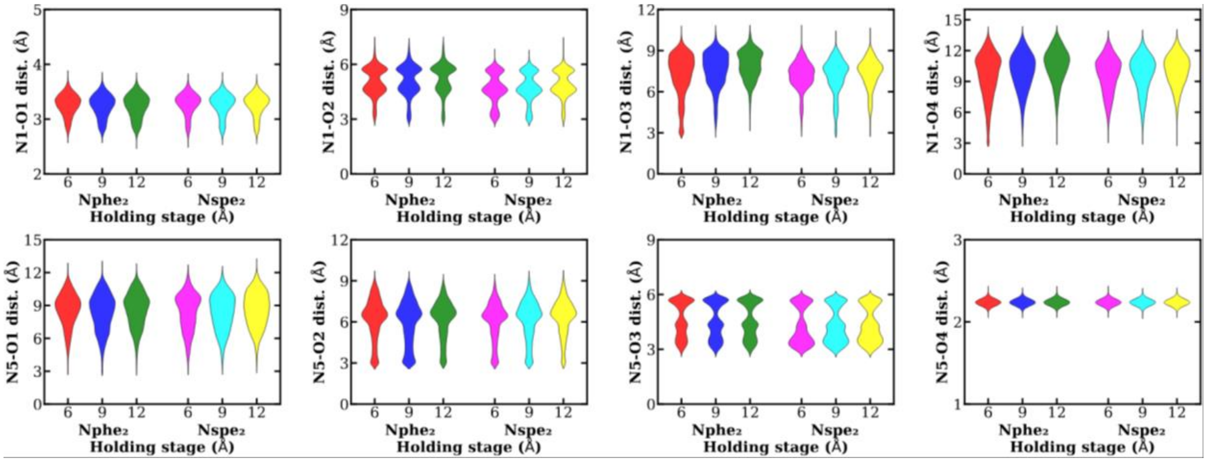

Fig. S24: Violin plots showing backbone H-bonding distance distribution for $Nphe_2$ and $Nspe_2$ for all possible donor-acceptor combinations. No differences are observed at different inter-anchor displacement potentials of 6 Å, 9 Å, and 12 Å holding stages indicating hydrogen bonding does not contribute significantly to the electron transport pathways.

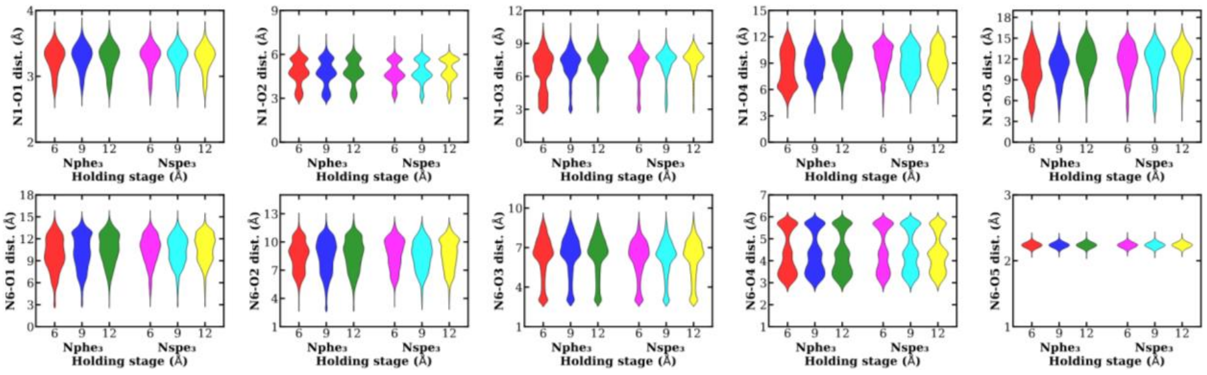

Fig. S25: Violin plots showing backbone H-bonding distance distribution for $Nphe_3$ and $Nspe_3$ for all possible donor-acceptor combinations. No differences are observed at different inter-anchor displacement potentials of 6 Å, 9 Å, and 12 Å holding stages indicating hydrogen bonding does not contribute significantly to the electron transport pathways.

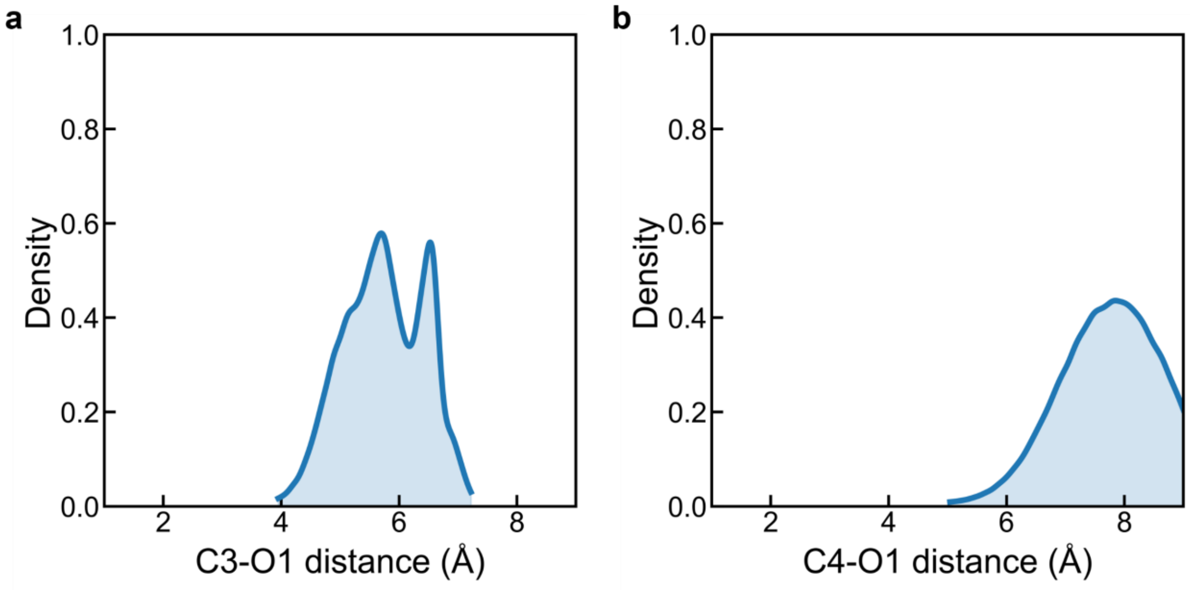

Fig. S26: Analysis of donor acceptor interactions between backbone methylene groups and carbonyl oxygens in $Nphe_2$, indicating the absence of hydrogen-bonding interactions.

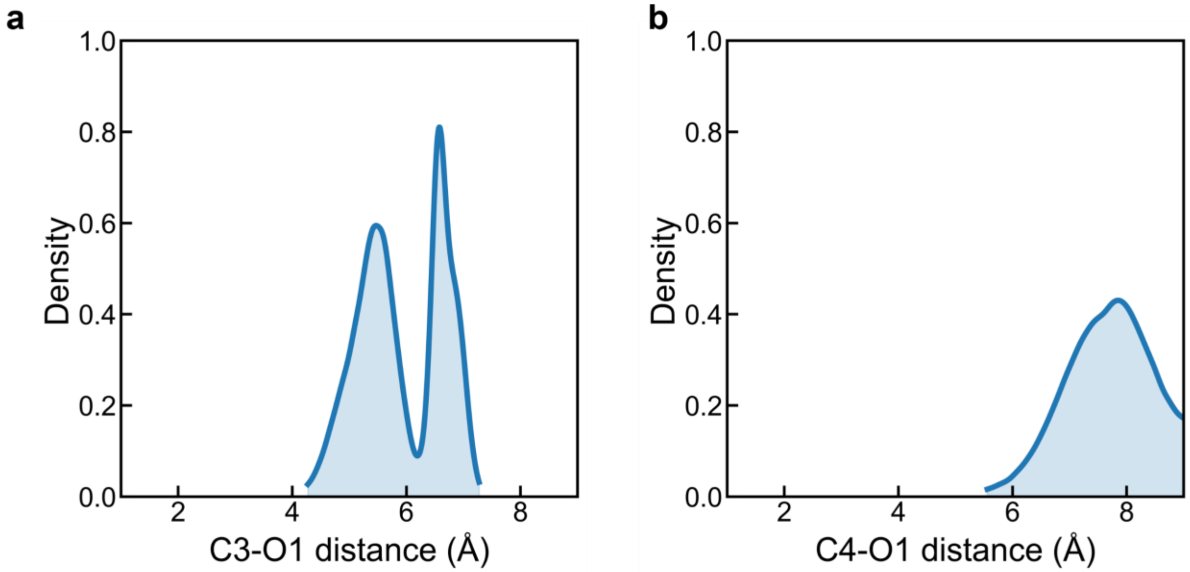

Fig. S27: Analysis of donor acceptor interactions between backbone methylene groups and carbonyl oxygens in $Nspe_2$, indicating the absence of hydrogen-bonding interactions.

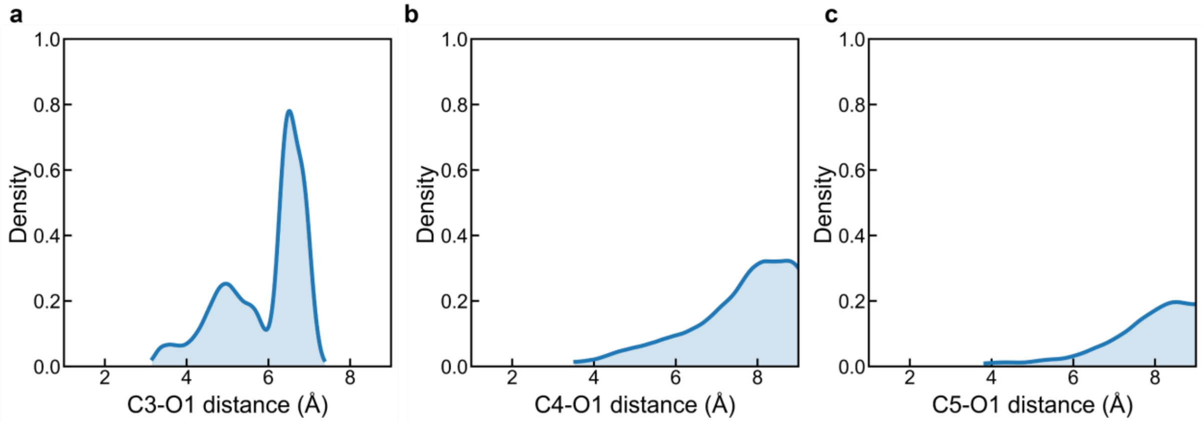

Fig. S28: Analysis of donor acceptor interactions between backbone methylene groups and carbonyl oxygens in $Nphe_3$, indicating the absence of hydrogen-bonding interactions.

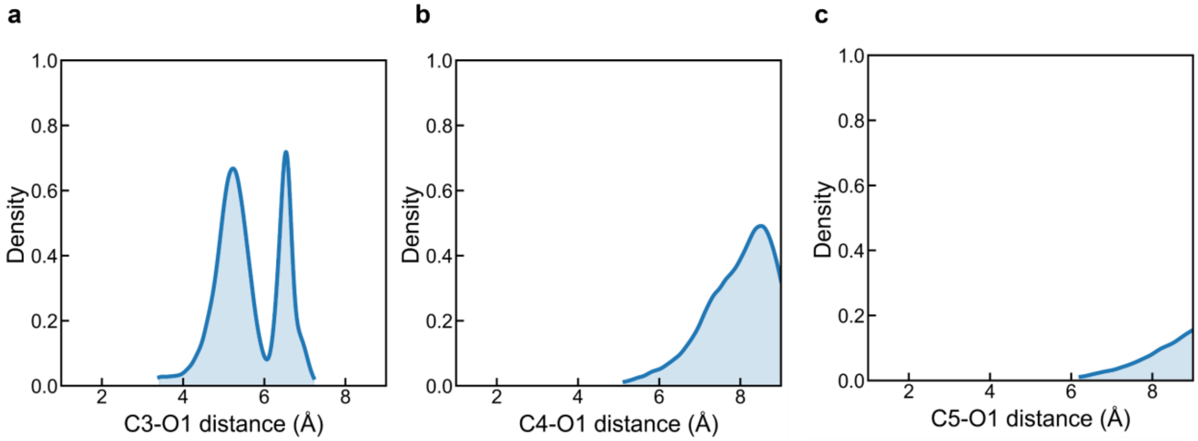

Fig. S29: Analysis of donor acceptor interactions between backbone methylene groups and carbonyl oxygens in $Nspe_3$, indicating the absence of hydrogen-bonding interactions.

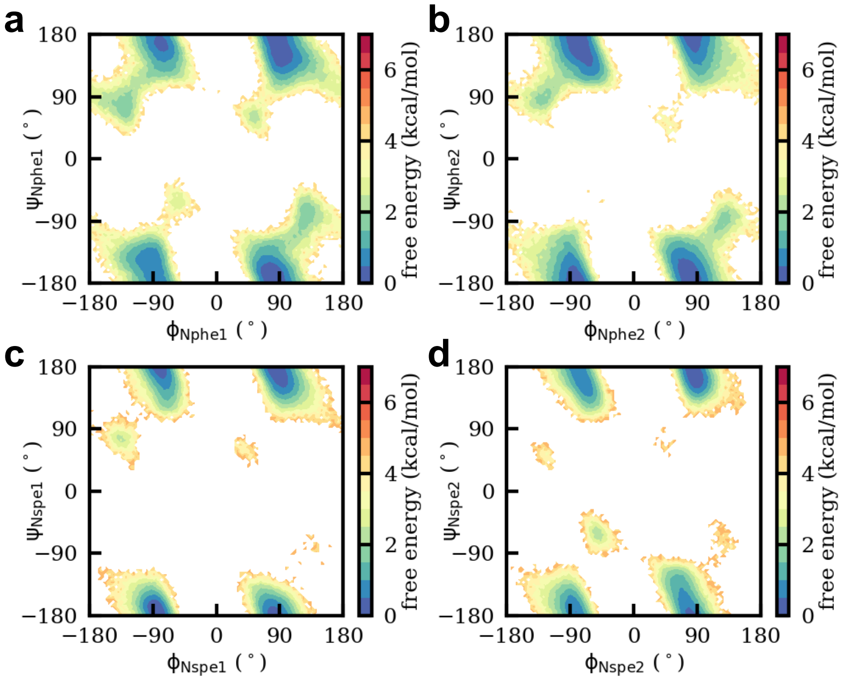

Fig. S30: Ramachandran free energy plots for peptoids (a), (b) $Nphe_2$ and (c), (d) $Nspe_2$. The left column (subfigures a and c) shows residue 1, and the right column (subfigures b and d) shows residue 2 (0-based residue indexing).

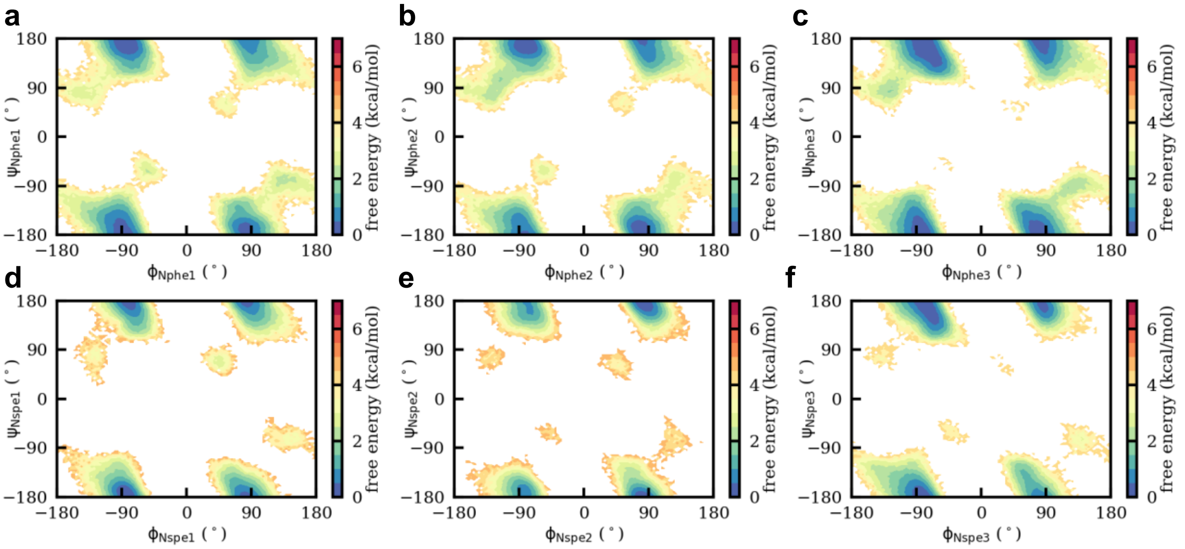

Fig. S31: Ramachandran free energy plots for peptoids (a), (b), (c) Nphe3 and (d), (e), (f) Nspe3. Left column shows residue 1 (subfigures a and d), middle column shows residue 2 (subfigures b and e), and right column (subfigures e and f) shows residue 3 (0-based residue index).

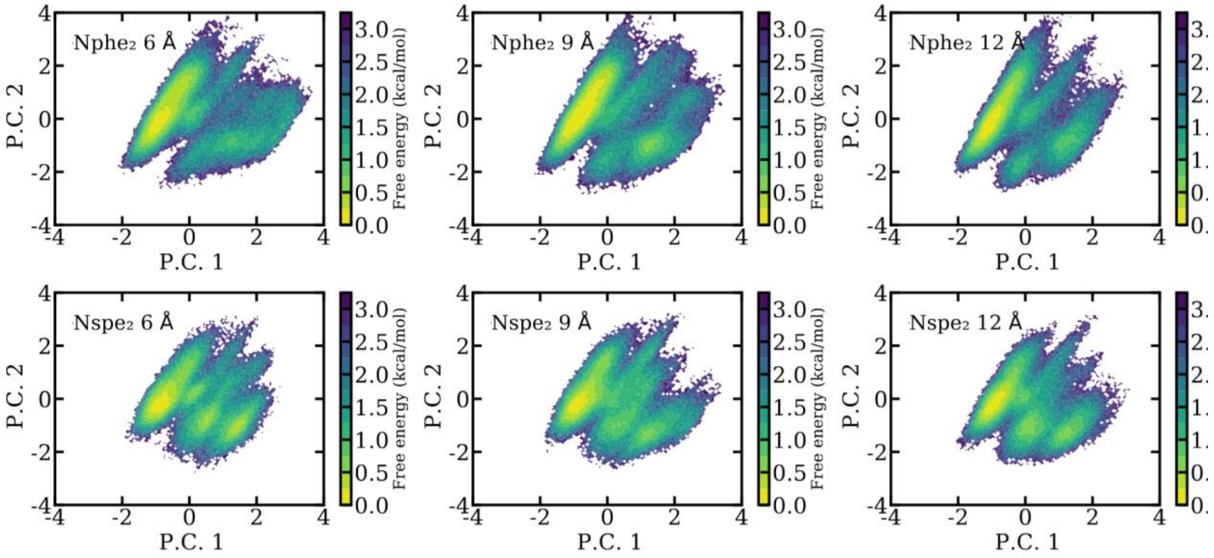

Fig. S32: Principal component analysis (PCA) for $Nphe_2$ and $Nspe_2$ at various inter anchor displacement potential (6 Å, 9 Å, and 12 Å).

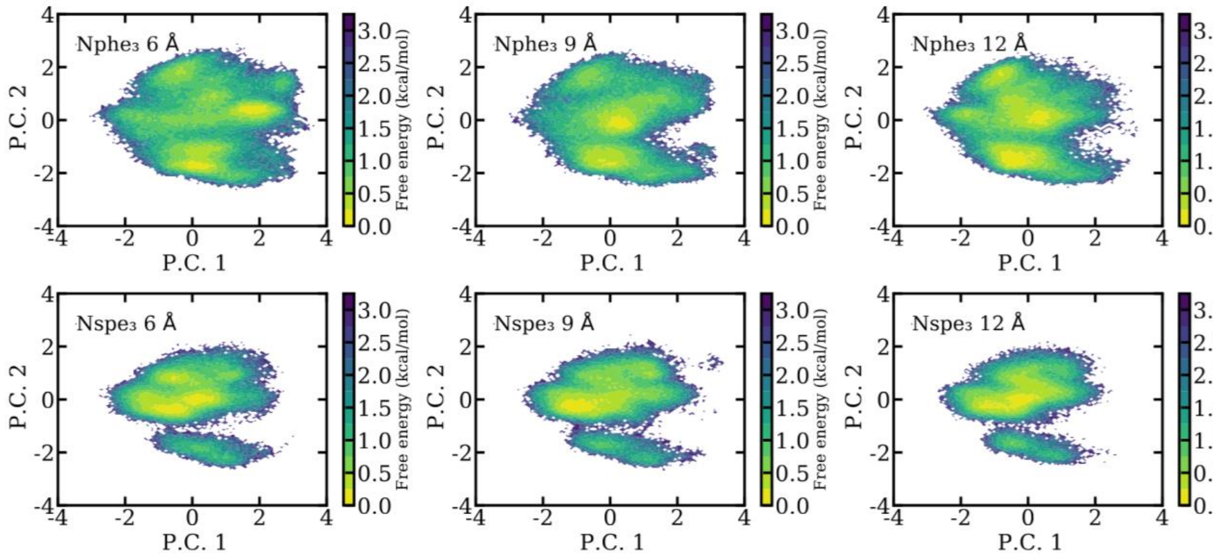

Fig. S33: Principal component analysis (PCA) for $Nphe_3$ and $Nspe_3$ at various inter anchor displacement potential (6 Å, 9 Å, and 12 Å). PCA results for $Nphe_3$ and $Nspe_3$ are inconclusive, likely due to the greater molecular length resulting in higher dimensionality, which limits PCA's ability to characterize features effectively.

S6. Non-equilibrium Green's function–density functional theory (NEGF-DFT) calculations

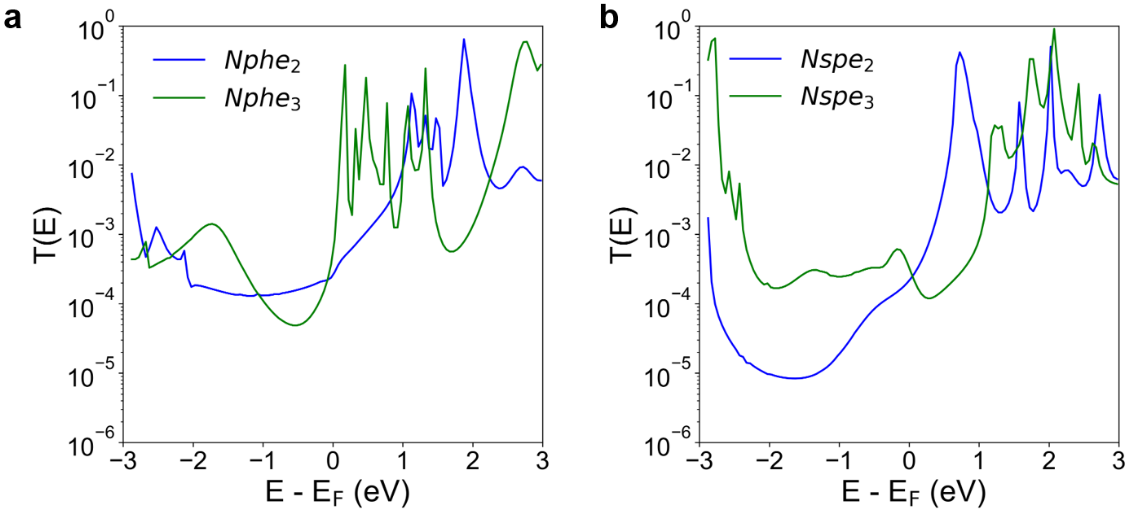

Fig. S34: Non-equilibrium Green's function-density functional theory (NEGF-DFT) calculations for $Nphe_3$ and $Nspe_3$.

Table S2: Conductance values at the Fermi energy level for peptoids

**Peptoid sequence**	**Electron transmission**
$Nphe_2$	2.6 X 10^{-4}
$Nphe_3$	5.9 X 10^{-4}
$Nspe_2$	2.16 X 10^{-4}
$Nspe_3$	3.3 X 10^{-4}

S7. Tunneling pathway model: Bond counting for pathway determination

In this section, we compare the electron tunneling pathways for peptides and peptoids. If the charge transport in peptides or peptoids is completely through-bond, then the transport is required between atoms 1→16 (**Fig. S35**). For the folded structures of peptides, corresponding to the high conductance state in molecular scale experiments, an H-bond occurs between atoms 5* and 12* (**Fig. S35a)**. In the case of electron transport through a hydrogen bond, transport can occur through 13 atoms [1→5 +5→5*+5*→12 (H-bond) + 12→16] as compared to 16 atoms.

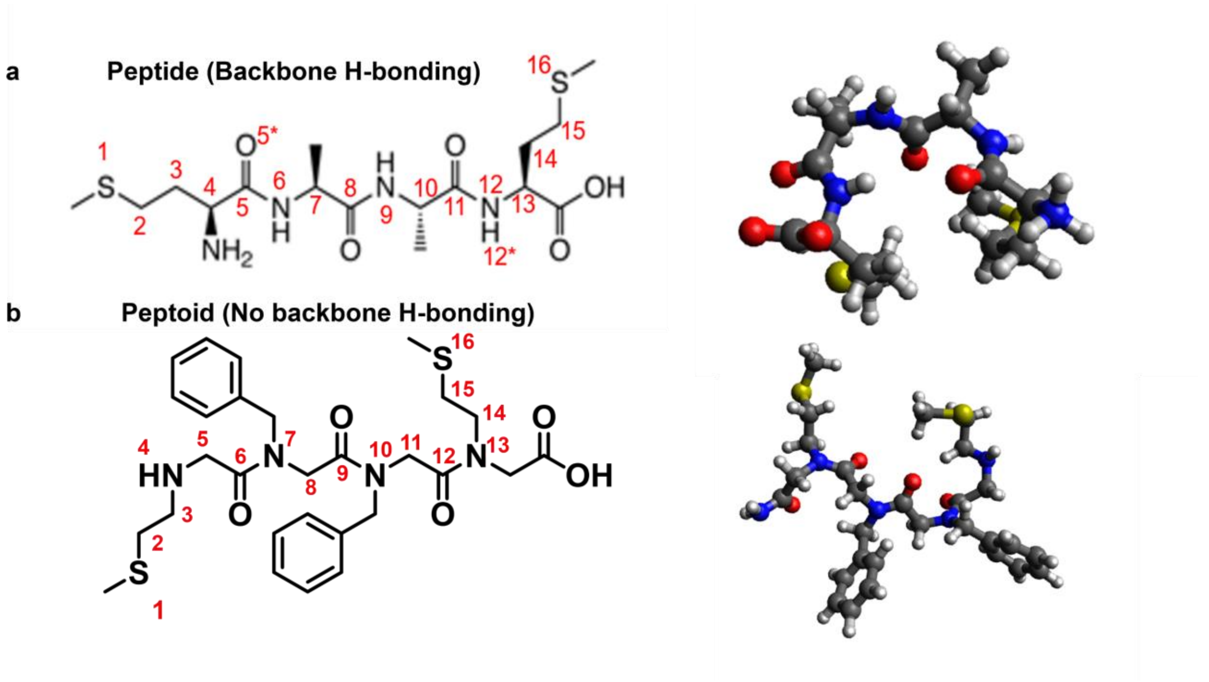

Fig. S35: Comparison between the tunneling pathways of peptides and peptoids. (a) Peptides contain backbone H-bonding and hence have a shorter path for electron transport. (b) Peptoids have no backbone H-bonding and hence have a longer pathway for electron transport. Analogous 3D structures of peptide and peptoid are also shown.

The conductance decay associated with a covalent bond (ϵ_c) and an H-bond (ϵ_H) are given by the following expressions: [2, 3]

$$\epsilon_c=0.6 \quad (1)$$

$$\epsilon_H= 0.36 \exp [-1.7(R - 2.8)] \quad (2)$$

For entirely through-bond mediated transport (1→16), electrons need to travel across 15 covalent bonds, yielding a conductance decay of $(0.6)^{15}$ **=** 0.00047. This conductance decay corresponds to electron transport in peptoids and the low-conductance state of

peptides, exhibiting values similar to those observed in molecular-scale break junction experiments.

On the contrary, if electron transport occurs via a combination of through-bond and through H-bond for peptides (1→5 +5→5*+5*→12 (H-bond) + 12→16), corresponding to the high conductance state observed in the molecular scale break junction experiments, electrons need to travel 9 covalent bonds and 1 H-bond. Here, the decay is $(0.6)^9(0.36 \exp[-1.7(R - 2.8)]) = 0.0026$ where R is approximately 3 Å for the MAAM sequence **(Fig. S35a**), as discussed above.

S8. References

1. Samajdar, R.; Meigooni, M.; Yang, H.; Li, J.; Liu, X.; Jackson, N. E.; Mosquera, M. A.; Tajkhorshid, E.; Schroeder, C. M. Secondary structure determines electron transport in peptides. *Proceedings of the National Academy of Sciences* **2024**, 121 (32), e2403324121. DOI: doi:10.1073/pnas.2403324121.
2. Beratan, D. N.; Betts, J. N.; Onuchic, J. N. Protein Electron Transfer Rates Set by the Bridging Secondary and Tertiary Structure. *Science* **1991**, *252* (5010), 1285-1288. DOI: 10.1126/science.1656523.
3. Betts, J. N.; Beratan, D. N.; Onuchic, J. N. Mapping electron tunneling pathways: an algorithm that finds the "minimum length"/maximum coupling pathway between electron donors and acceptors in proteins. *Journal of the American Chemical Society* **1992**, *114* (11), 4043-4046. DOI: 10.1021/ja00037a004.
